# Spatio-Temporal Analyses of *Symbiodinium* Physiology of the Coral *Pocillopora verrucosa* along Large-Scale Nutrient and Temperature Gradients in the Red Sea

**DOI:** 10.1371/journal.pone.0103179

**Published:** 2014-08-19

**Authors:** Yvonne Sawall, Abdulmohsin Al-Sofyani, Eulalia Banguera-Hinestroza, Christian R. Voolstra

**Affiliations:** 1 Benthic Ecology group, Helmholtz Center for Ocean Research (GEOMAR), Kiel, Germany; 2 Department of Marine Biology, Faculty of Marine Sciences, King Abdulaziz University (KAU), Jeddah, Saudi Arabia; 3 Red Sea Research Center, King Abdullah University of Science and Technology (KAUST), Thuwal, Saudi Arabia; University of California- Santa Barbara, United States of America

## Abstract

Algal symbionts (zooxanthellae, genus *Symbiodinium*) of scleractinian corals respond strongly to temperature, nutrient and light changes. These factors vary greatly along the north-south gradient in the Red Sea and include conditions, which are outside of those typically considered optimal for coral growth. Nevertheless, coral communities thrive throughout the Red Sea, suggesting that zooxanthellae have successfully acclimatized or adapted to the harsh conditions they experience particularly in the south (high temperatures and high nutrient supply). As such, the Red Sea is a region, which may help to better understand how zooxanthellae and their coral hosts successfully acclimatize or adapt to environmental change (e.g. increased temperatures and localized eutrophication). To gain further insight into the physiology of coral symbionts in the Red Sea, we examined the abundance of dominant *Symbiodinium* types associated with the coral *Pocillopora verrucosa*, and measured *Symbiodinium* physiological characteristics (i.e. photosynthetic processes, cell density, pigmentation, and protein composition) along the latitudinal gradient of the Red Sea in summer and winter. Despite the strong environmental gradients from north to south, our results demonstrate that *Symbiodinium microadriaticum* (type A1) was the predominant species in *P. verrucosa* along the latitudinal gradient. Furthermore, measured physiological characteristics were found to vary more with prevailing seasonal environmental conditions than with region-specific differences, although the measured environmental parameters displayed much higher spatial than temporal variability. We conclude that our findings might present the result of long-term acclimatization or adaptation of *S. microadriaticum* to regionally specific conditions within the Red Sea. Of additional note, high nutrients in the South correlated with high zooxanthellae density indicating a compensation for a temperature-driven loss of photosynthetic performance, which may prove promising for the resilience of these corals under increase of temperature increase and eutrophication.

## Introduction

Coral reefs do not typically exist at temperatures greater than 31°C mean summer temperatures. Temperatures exceeding this upper limit cause damage to the photosynthetic apparatus of zooxanthellae [1], the endosymbiotic dinoflagellates (genus *Symbiodinium*) of corals, which are essential for coral health and survivability [2], [3]. Numerous studies have shown that the ‘inactivation’ of zooxanthellae in consequence of increased temperatures, lead to a loss of zooxanthellae, finally resulting in coral bleaching (see review and commentary [4], [5]). Although the temperature thresholds for the onset of coral bleaching have been shown to vary geographically due to localized adaptations [4], diverse and flourishing reefs above 31°C are rare, but present in the southern Red Sea [6], [7]. This suggests that the zooxanthellae in the southern Red Sea may have acclimatized or undergone adaptations, which allow them to cope with increased temperatures. The ability to cope with elevated temperatures is of particular interest in the light of climate change scenarios, which predict an increase of 1–3°C for tropical ocean temperatures over the next several decades [8], [9].

In addition to predicted temperatures increases, nutrient loading to coastal systems is also expected to increase over the next decades due to ongoing coastal urbanization and land-use activities [10]. While nutrient enrichment has clearly negative effects on coral reef ecosystems, zooxanthellae initially profit from higher nutrient supplies, which increases their number [11]–[13], and/or their pigmentation and protein content [11]. However, negative effects on the coral host may occur. For instance, the coral host can lose control over the nutrient supply to the zooxanthellae, which can lead to an uncontrolled increase of zooxanthellae numbers and further to a disruption of substrate recycling between host and symbionts [11], [14]. Further, unbalanced nutrient ratio supplies (nitrogen:phosphor) can lead to higher stress-sensitivity of zooxanthellae [15]. Additionally, increased nutrients can lead to an accumulation of particulate organic matter, which reduces light penetration through the water column [16]. Zooxanthellae can acclimatize to decreasing light intensities to a certain degree by increasing their concentration of photo-collecting pigments (e.g. chlorophyll *a* and *c*2) and photosynthesis associated proteins [11], [17], [18] and by a decrease of photo-protective pigments (xanthophyll) [19], [20].

Acclimatization potential of zooxanthellae was also shown to vary between different *Symbiodinium* types featuring different physiological strengths and susceptibilities to environmental changes [21], [22], of which the exact correlation is not clear. However, it has been suggested that certain types belonging to *Symbiodinium* clade D are rather heat-tolerant and are present in particular at near-shore locations or reefs flat frequently exposed to air [23], [24] and after heat-stress events in the Indo-Pacific [25], [26]. Other studies suggested a high level of host symbiont specificity, which may imply a high acclimatization potential or local adaptation of a given clade type (reviewed by Goulet [27]).

While the sole effects of temperature and nutrients on zooxanthellae (and their hosts) are well understood, the combined impacts of high nutrients and temperatures under natural conditions are poorly understood. Given that nutrient loading to coastal systems is expected to occur in parallel with temperature increases over the next decades, it is essential to understand the combined effects of higher temperatures and nutrient concentrations on the coral holobiont [28] and in particular on zooxanthellae, and to determine how they respond to these conditions.

The limited data available suggest that increased availability of food enhances the nutritional status of corals, which further supports zooxanthellae functioning and thereby decreases bleaching susceptibility, although the actual mechanisms need to be further understood [29], [30]. Further, higher zooxanthellae densities in corals increased bleaching susceptibility, although zooxanthellae numbers were not related to nutrient supply, but rather to species-specific differences [31].

The Red Sea proper (defined as the part of the Red Sea without the Gulf of Suez and Aqaba), which until now is an under-investigated area in terms of coral biology, provides an ideal model system in which to examine the potential of zooxanthellae to acclimatize or adapt to a combination of high temperatures and nutrient concentrations. Nutrient-rich waters enter the Red Sea from the Indian Ocean through the Bab el Mandeb in the south [32], and lead to chlorophyll *a* (chl *a*) concentrations as high as 4.0 µg l^−1^ in the southern Red Sea. Within the same region, atmospheric warming can lead to sea surface temperatures up to 33°C. Towards the north, temperature decreases, nutrients are gradually depleted, and salinity increases (due to decreasing atmospheric temperature, low fresh water inflow, and high evaporation). Although the prevailing environmental conditions in the southern Red Sea are considered marginal for coral growth [33], [34], corals are abundant in this region and species richness is only slightly lower than in the north (∼115 vs. 187 described hermatypic species [6]). In the north, temperatures and nutrient concentrations are lower and more characteristic of those typically considered optimal for zooxanthellae and coral health.

In order to examine acclimatization/adaptation of zooxanthellae under high temperature and nutrient conditions in the southern Red Sea, the photobiology and genetic variability of *Symbiodinium* associated with the widely distributed Red Sea coral *Pocillopora verrucosa* was investigated along this north-south temperature and nutrient gradient in a seasonal manner.

## Materials and Methods

### Ethics Statement

The research permission was given by the Minister of Higher Education and the Ministry of Defense, Department of Marine Survey 273 in Saudi Arabia. The sampled reefs do not fall under any legislative protection or special designation as a marine/environmental protected area. No special permit is required for the sampled reefs and organisms. The Saudi Coastguard Authority issued sailing permits to the sites that included coral collection. *Pocillopora verrucosa* is listed as ‘least concern’ on the ICUN Red List (http://www.iucnredlist.org). The CITES permission number is 11-SA-0197-PD. Import documents for Germany were not required for frozen coral tissue slurry.

### Study species and study sites

**Figure 1:**
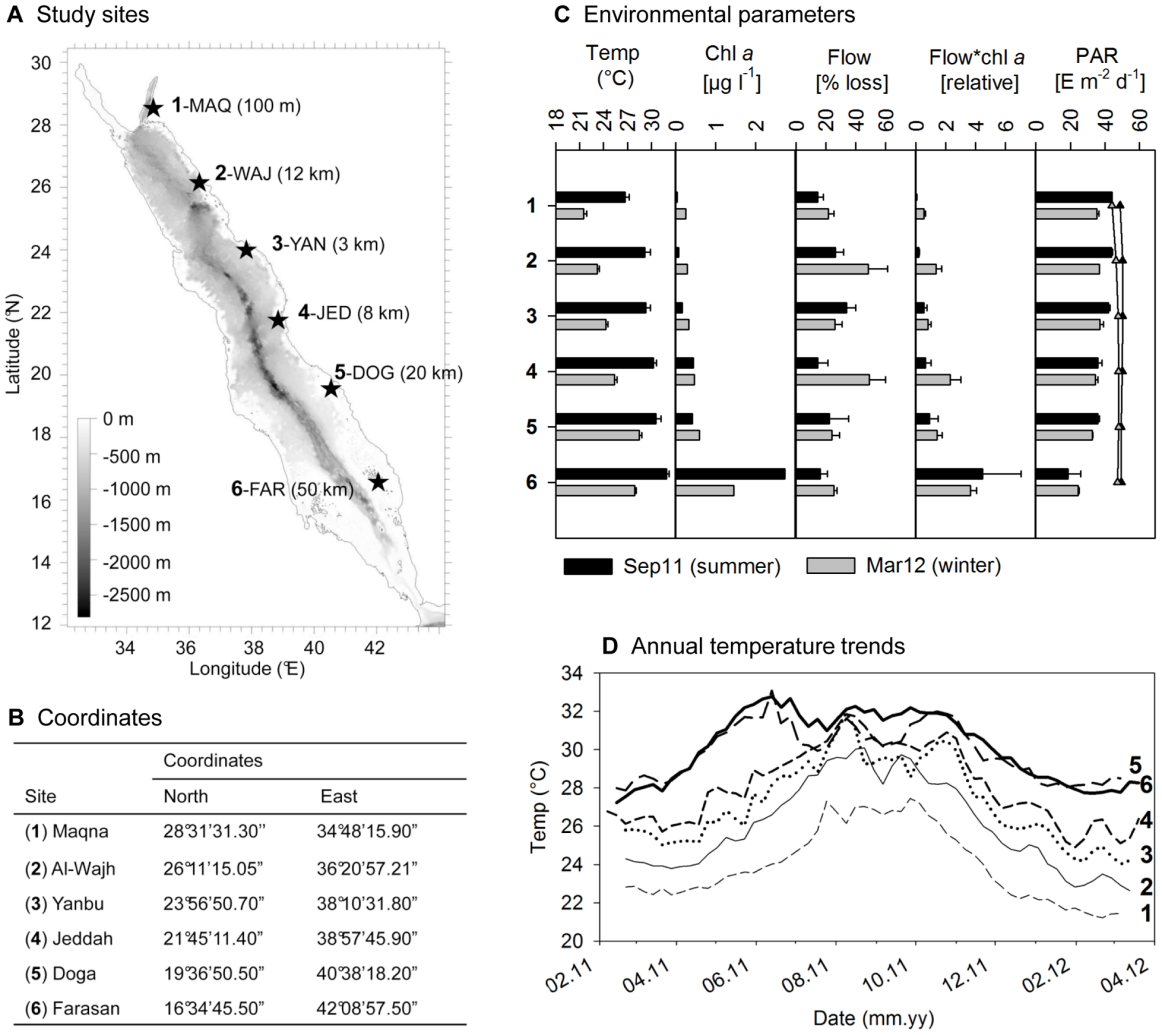
Study sites and environmental conditions in the Red Sea. Sites are labeled from 1 to 6 from north to south. (**A**) Map with study sites and distance from shore in brackets. At site 6, distance from the main land is 50 km, but only 500 m from the island. (**B**) Coordinates of the study sites. (**C**) Environmental conditions at study sites (1 to 6) during summer and winter presented as mean ± SD. Photosynthetic active radiation (PAR) is also presented for the ocean surface (triangles) in order to demonstrate the effect of nutrients (chl *a*) on light attenuation in 5 m depth. Flow*chl *a* is a relative measure of nutrient flux, for which flow (% gypsum loss) was multiplied by chl *a* concentration. (**D**) Temperature trends at study sites (1–6) from February 2011 to March 2012 in 5 m depth.

The widely distributed and common coral species *Pocillopora verrucosa* (Ellis and Solander, 1786) was chosen for the study and identified after Veron [35]. It is found throughout the Red Sea, particularly in shallow, high light areas, in exposed reef fronts, as well as protected back reefs [35]. Highest abundances are found in the northern and central Red Sea proper, while it becomes less abundant towards the south [7]. Bleaching susceptibility was found to be rather low compared to other species [36]. Investigations took place at exposed reef fronts in 5 m depth at 6 sites along the Saudi Arabian Red Sea coast between the latitudes 16°34′ and 28°31′N (Fig. 1). The reefs were at least 3 km off the coast, to avoid potential land-based influences as much as possible. One exception is given in the Gulf of Aqaba (1-MAQ), where reef structures only exist along the coast line. Here, the selected reef was approximately 10 km away from the next human settlement, but 100 m off-shore.

### Environmental parameters

Temperature loggers (HOBO Pendant, Onset USA) were deployed in 5 m depth from February 2011 until March 2012 and monthly temperature averages were calculated for the two sampling months, September 2011 (Sep11) and March 2012 (Mar12).

In order to evaluate the relative exposure to water flow, plaster balls were produced with commercial gypsum after the principle described by Jokiel and Morrissey [37]. For this, tennis balls were filled with gypsum and a wooden stick was inserted to hold the plaster balls. The tennis balls were removed after a few hours, the gypsum balls were dried for several days, then weighed and finally fixed to a metal rod previously hammered into the reef (n = 5 in 3–6 m depth). One plaster ball served as a control and was placed into a bucket in order to assess the dissolution of plaster without water flow. After 48 h the plaster balls were retrieved, dried and weighed again. The loss of weight (minus control) served as a relative measure of water flow.

Chlorophyll *a* (chl *a*) concentrations, light attenuation coefficients (K_d_) and intensity of photosynthetic active radiation (PAR) at the water surface were derived from satellite derived data sets of NASA, Giovanni online data system, Ocean Color Radiometry, monthly averaged MODIS-Aqua 4 km, for the corresponding season and year. Chl *a* and K_d_ values are derived by remote sensing of inherent optical properties of the water body, where the absorption, scattering and backscattering of specific spectral bands were used to calculate the chl *a* concentration, and the total of spectral bands together with the mean vertical diffuse attenuation coefficient over the first optical depth were used to calculate the K_d_ value [38], [39]. The raw data is presented as Supporting Information (Table S1).

PAR intensity for the experimental depth of 5 m was calculated with the equation K_d_ = ln(PAR_5m_/PAR_surface_)/5 [40]. In order to approximate the actual nutrient availability to the corals, the relative nutrient flux was calculated by multiplying the chl *a* concentration, a commonly used proxy for nutrient loads in reef environments [16], [34], by water flow (flow*chl *a*). Since the water flow is only a relative measure expressed in % loss of gypsum weight, nutrient flux is a relative measure, as well. A possible limitation of the flow data is the representation of only 2 days, in contrast to the other environmental data, which represent the conditions of the entire experimental month.

### Experimental design and metabolism measurements

Prior to incubations for metabolism measurements, 6 coral fragments (∼5–7 cm long branches) from 5 m depth were chiseled off from the central part of 6 colonies (1 fragment per colony, minimum distance between colonies 5 m) at each site. Each fragment was glued to a plastic screw with under water epoxy (ORCA Construct, Aquarium Muenster, Germany) and the screws were fixed to a basket installed at the sea floor upside down. The fragments were acclimatized for one day. They appeared to be in good condition prior incubations as indicated by behavioral comparison to neighboring colonies (i.e. polyps were extended). Three coral fragments were placed in 3 incubation chambers (1 fragment per chamber) from 0800/0900 to 1600/1700 hrs, which were deployed at the experimental depth. One additional chamber served as a coral-free control. For respiration measurements the chambers were darkened by covering the entire setup with a black cloth over one incubation interval in the morning (45 min) (for further explanation of the incubation set up see paragraph below). The incubations were repeated the next day with new coral fragments, resulting in 6 replicates per site. Dark respiration rates were calculated and Photosynthesis-Irradiance (P-I) curves were constructed. A curve was fitted through the data points using the software SigmaPlot 10.0 and the “Regression Wizard” with the equation category “Exponential rise to maximum” and the equation y = a(1−e^−bx^), where y is P_n_ at a given light intensity, a is P_n_(max), b is initial slope/P_n_(max) and x is the given light intensity [41]. The net photosynthetic rate at PAR 600 µmol photons m^−2^ s^−1^ (P_n_(600)) was derived from the P-I curves.

### Set up of *in situ* incubations with corals

An *in situ* incubation setup was constructed at the GEOMAR (photograph presented as Fig. S1): Each of the 4 cylindrical acrylic chambers (volume: 950 ml) contained a battery-run stirrer (low speed), a water inlet and outlet with one-way valves in front and behind the chamber respectively, a fragment holder and an oxygen sensor (DIGISENS-Optod, Ponsel, France). All 4 oxygen sensors were connected to a battery-run data logger (SDL500 Submersible Data Logger, NexSens Technology, USA), which logged the oxygen concentration every minute. An UW-PAR sensor (Li-192, LiCor Biosciences GmbH, Germany) was installed next to the chambers at the same height and connected to the same data logger. PAR was logged simultaneously with the oxygen measurements. Each water inlet was connected to a programmable battery-run pump via tubing, which pumped the surrounding water through the chambers every 45 min for 2 min to flush the chambers. All components of the setup were fixed to a POM (Polyoxymethylen) frame for easy and robust handling.

### Fluorescence measurements

Photochemical measurements were taken based on the chl *a* fluorescence properties with a pulse amplitude modulation fluorometer (Diving-PAM, Walz, Germany) [29], [42]. After incubations, the fragments were brought to the laboratory and allowed to recover from transport for ∼5 h in aquaria filled with water from the site of origin. Fragments were dark adapted for 30 min and the maximum quantum yield (F_v_/F_m_) was measured. The fragments were again dark adapted for 30 min and rapid light curves (RLC) were performed. The PAR intensities for the RLCs were increased every 20 s from 0 to 1140 µmol photons m^−2^ s^−1^ over 8 steps. Non-photochemical quenching (NPQ: [F_m_/F_m_′]−1), a measure of the thermal dissipation of the excess absorbed excitation energy, was calculated and plotted against PAR. NPQ at PAR 600 µmol photons m^−2^ s^−1^ (NPQ (600)) was calculated after fitting the same curve as for the P-I curves.

### Measurement of *Symbiodinium* properties and coral surface area

After the fluorescence measurements, the coral tissue was removed from the skeleton with an air gun and filtered seawater (0.7 µm). The volume of the tissue slurry was measured (20–45 ml) and homogenized with a T18 basic Ultra Turrax (IKA, Staufen, Germany; 10 s, 15,000 U min^−1^) in order to break open coral cells and release zooxanthellae from coral tissue. Light microscopy for a subset of samples confirmed the presence of intact zooxanthellae. Sub-samples were stored at −20°C until zooxanthellae density and protein analyses, at −80°C until pigment analyses and in RNA*later* (Invitrogen) for genetic analyses (*see* clade identification). Zooxanthellae of defrosted samples were counted with a hemocytometer (Fuchs-Rosenthal chamber) under the light microscope to determine zooxanthellae density (6 replicate counts). For protein analyses, animal tissue and zooxanthellae were separated by centrifugation (5 min, 9,000 rpm, 4°C), the zooxanthellae pellet was washed by dissolving the pellet with filtered seawater, subsequent centrifugation (2 repetitions), and finally dissolved in distilled water containing 1% sodium dodecyl sulfate (SDS) to open the zooxanthellae cells for intracellular protein release. Protein concentrations were determined photometrically (DU 650 Spectrophotometer, Beckman, USA) using the BCA Protein Assay kit (Thermo Scientific, Rockford) with bovine serum albumin as a standard. The photo-collecting pigments chl *a*, *c2* and peridinin, and the photo-protecting pigments diadinoxanthin and diatoxanthin (summarized as xanthophyll [xantho]) were extracted from the zooxanthellae with 100% acetone (24 h, 4°C). Pigments were analyzed by HPLC and separated in a Varian Microsorb-MV column (100-3, C8, 100×4.6 mm) with 70% MeOH/30% 1M NH4-acetate and 100% MeOH as eluents. Pigments were identified and quantified using both a fluorescence detector (Waters 474 Scanning Fluorescence Detector, Waters GmbH) and a photodiode array absorption detector (Waters 2996 Photodiode Array Detector).

The surface area of the coral fragments was determined gravimetrically using the wax coating technique [43]. Briefly, 2 times the coral fragments were dipped into melted wax (kept at constant temperature) for 2 seconds, dried and weighted. The weight gain between the first and second dipping was related to the surface area, which was previously determined by a standard curve. The standard curve was done with a serious of wooden cubes of known surface area, following the same procedure (linear regression R^2^ = 0.993).

### *Symbiodinium* type identification

For *Symbiodinium*-typing, DNA was extracted using the DNeasy Plant Mini Kit (Qiagen, Hilden, Germany) according to the manufacturer's instructions. The ITS2 rDNA region was amplified with the primer pair ITSintfor2 and ITS2CLAMP [44] using PCR conditions described in LaJeunesse et al. [45]. Amplified fragments were separated on 8% polyacrylamide gels, following Sampayo et al. [46], and using a CIPHER DGGE KIT (CBS Scientific Company, Del Mar, CA). Gels were run at 150 V for 15 h and stained for 20 min with 1× SYBR Green (Invitrogen, Carlsbad, CA) and visualized on a Dark Reader Transilluminator (Clare Chemical Research, Dolores, CO).

*Symbiodinium* types were determined by DGGE fingerprint profiles and sequencing. For sequencing, prominent bands that are representatives of the dominant ITS2 type in a sample were excised from the DGGE gel and re-amplified as described in LaJeunesse [47] (example gels are provided as Fig. S2). Re-amplified products were purified following manufacturer's instructions for Illustra ExoStar (GE Life Sciences, Piscataway, NJ). Samples were sequenced in both, forward and reverse directions at the KAUST BioScience Core Lab (Thuwal, Saudi Arabia). Sequences were trimmed and assembled into contigs using Chromas-pro v. 1.5 (free trial available at http://technelysium.com.au/) and exported to MEGA program V.5 [48] where alignments were constructed using MUSCLE [49]. Each individual contig was aligned with ITS2 sequences downloaded from the GeoSymbio database [50]. ITS2 types were designated according to similarity to sequences from the GeoSymbio database and the DGGE fingerprint profile. If the DGGE profile indicated ITS2 bands that ran as background bands to similar main bands, the ITS2 band/sequence was named according to the main band with the next available alphabetic character suffix. If the DGGE profile produced ITS2 background bands that were distinct to the sequence of the main band, or if the DGGE profile produce a single specific band that was different from available main bands, the ITS2 band/sequence was named according to the most similar clade with the next available numeric character. New ITS2 types were submitted to the genbank (NCBI) and have the following accession numbers: KF939531 (C1oo), KF939533 (C1nn), F939530 (C85), KF939534 (A21).

### Data analysis

Multivariate statistics were performed with the software PRIMER v6 and the add-on software PERMANOVA+ [51]. Initially, trends within the complete data set of zooxanthellae characteristics, the properties (pigment concentrations and ratios, protein concentration and cell density) and performance (photosynthetic rates, F_v_/F_m_, NPQ), were explored by principal coordinate analyses (PCO) an equivalent to principal component analyses (PCA), but with a higher flexibility of resemblance measures [51]. A resemblance matrix was created based on chi-squared distances, since non-linear relationships between some variables were expected, and samples were projected on PCO axes that minimize residual variation in the space of the resemblance matrix.

Next, the potential relationships between the zooxanthellae characteristics and the communities of dominant *Symbiodinium* types and between zooxanthellae characteristics and environmental factors were explored using the DISTLM routine of PERMANOVA+. This routine allows a variation partitioning according to a regression model exploring relationships between one or more predictor variables and the response variables represented as the PCO axes [51]. The distance measure for the clade composition was Euclidean distance and for the zooxanthellae characteristic chi-squared distance.

The potential effects of environmental conditions (temperature, nutrients [flow*chl *a*] and light) on individual zooxanthellae properties (i.e. zooxanthellae density, concentration of photo-collecting pigments cell^−1^, protein cell^−1^ and xantho/chl *a* ratio) were tested by regression analyses using STATISTICA 8. Analyses were conducted with complete data sets (all sites and both seasons) and seasonal sub-sets (Sep11 or Mar12) to differentiate between overall patterns across sites and seasons and pure geographic patterns (north-south), respectively. Furthermore, the comparison of the results of the complete data set and the seasonal sub-sets allowed discriminating between seasonal and geographic patterns. Partial correlations were conducted to assess the ‘effect’ of each environmental parameter alone, while stepwise forward regression was used to assess possible combined ‘effects’ of environmental parameters. In case of a correlation between two environmental parameters with r^2^>0.5, only one of the two parameters was included into partial correlation and stepwise forward regression analyses. Forward step-wise regression was only performed when at least 2 parameters correlated with r^2^<0.5.

## Results

### Environmental parameters

Temperature at 5 m depth showed a spatial and temporal gradient (Fig. 1C, 1D). Temperature increased from north to south, while the trend was stronger during winter (Mar12, coldest month: 21.5±0.4°C to 28.5±0.3°C) than in summer (Sep11, end of warmest period: 26.7±0.5°C to 31.9±0.4°C). Seasonal fluctuation was highest in 2-WAJ with 6.0°C difference and lowest in 5-DOG with 2.1°C difference. Water flow (Fig. 1C) was generally higher in the northern and central Red Sea proper compared to the Gulf of Aqaba (1-Maqna) and the southern Red Sea (5-DOG, 6-FAR) as indicated by the % loss of gypsum from the gypsum balls after 48 h, and it was higher in Mar12 compared to Sep11. The NASA-derived chl *a* concentrations (Fig. 1C) were particularly high at the southern-most reef (6-FAR) with the highest monthly average value of 2.73 µg l^−1^ in Sep11. All other sites featured chl *a* concentrations below 0.60 µg l^−1^ during the experimental months, while they increased from north to south and were higher in Mar12 compared to Sep11, except at the most southern reef. Hence, nutrient flux (Fig. 1C) derived by the multiplication of flow and chl *a* concentration (flow*chl *a*) was also higher in Mar12 compared to Sep11. However the pattern of an extreme increase of chl *a* concentrations at 6-FAR, was reduced in the nutrient flux pattern, where due to comparatively low water flow the nutrient flux was lowered as well. The NASA-derived light intensities (PAR) were comparatively homogenous at the sea surface over site and season, but decreased moderately from 44.1 to 36.1 Einstein m^−2^ day^−1^ from north (1-MAQ) to south (5-DOG) with a sudden drop to 18.6 Einstein m^−2^ day^−1^ at the most southern reef in Sep11. The north-south trend was less pronounced in Mar12 (Fig. 1C).

The following correlations between the environmental parameters were found: a strong negative correlation between PAR and chl *a* concentration (R = −0.92, p<0.05), and between PAR and nutrient flux (R = −0.91, p<0.05), a weak positive relationship between temperature and chl *a* concentration (R = 0.35, p<0.05), and no relationship between temperature and nutrient flux (R = 0.16, p>0.05). Consequently, we used only the weakly or non-related parameters, namely temperature, flow and nutrient flux, in the multivariate analyses. Similarly, we used only temperature and nutrient flux or temperature and light in the partial correlation or multiple regression analyses, depending on the hypothesis.

### Abundance of dominant *Symbiodinium* types

**Figure 2:**
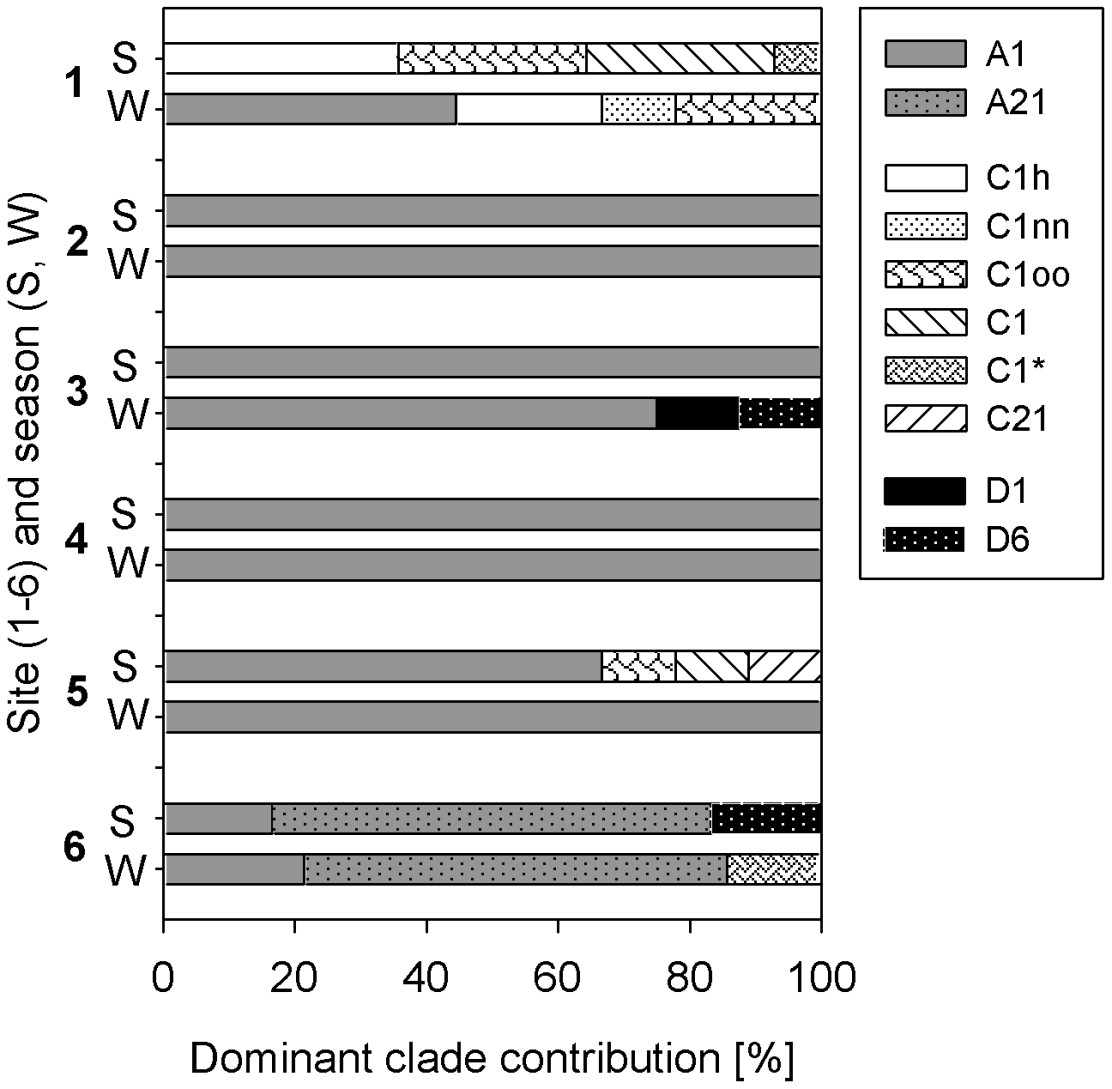
Composition of dominant *Symbiodinium* clades at all sites from north (1) to south (6) in summer (S, Sep11) and winter (W, Mar12). The rare and new ITS2 sequence found at 1-MAQ named “C85” is not included in the graph.

Zooxanthellae community of *P. verrucosa* was dominated by *Symbiodinium microadriaticum* (type A1, National Center for Marine Algae and Microbiota, [52]) throughout the range from 2-WAJ to 6-FAR (Fig. 2). Divergence from association with *S. microadriaticum* was found, however, particularly at 6-FAR, where ITS2 DGGE profiles produced a single main band that was distinct from type A1. The new type referred to as A21 deviated by 2 base pairs to type A1 (one transition and one transversion at 6-FAR) and warrants further investigation as it was only present at this site. Divergence from type A1 was also found at 3-YAN in one sample, where the ITS2 sequence deviated from type A1 by 1 base pair (one transversion). ITS2 types of clade C occurred mainly in the northernmost part of the Red Sea (1-MAQ) with mainly type C1h, but also type C1 (*S. goreaui*, [53]). Lower abundances of clade C types were found at 5-DOG in Sep11 and at 6-FAR in Mar12 in combination with dominant clade A types (mainly *S. microadriaticum*, Fig. 2). Most DGGE band profiles showed multiple bands of clade C types in individual coral samples (see Fig. S2). Sequencing of the different bands revealed differences in 1 or 2 base pairs in these ITS2 sequences compared to the ITS2 type C1 from GeoSymbio. These new ITS2 variants were named as C1nn (1 base pair different) and C1oo (2 base pairs different). In one instance a new clade C ITS2 sequence was found at 1-MAQ, which was differed from type C1 by a deletion of 16 base pairs, and is referred to C85. Finally, ITS2 type D6 was found in combination with *S. microadriaticum* at 3-YAN and at 6-FAR and type D1 at 3-YAN (Fig. 2). We found little seasonal variation overall. However, 4 out of the 6 sites displayed some levels of clade composition variation (<25% in the Red Sea proper and 45% in the Gulf of Aqaba) (Fig. 2). Since the designation of ecotypes from ITS2 sequence divergence is not straightforward, we remained conservative in our statistical analyses by differentiating between the main clades only, namely clade A, C and D.

### *Symbiodinium* characteristics

**Figure 3:**
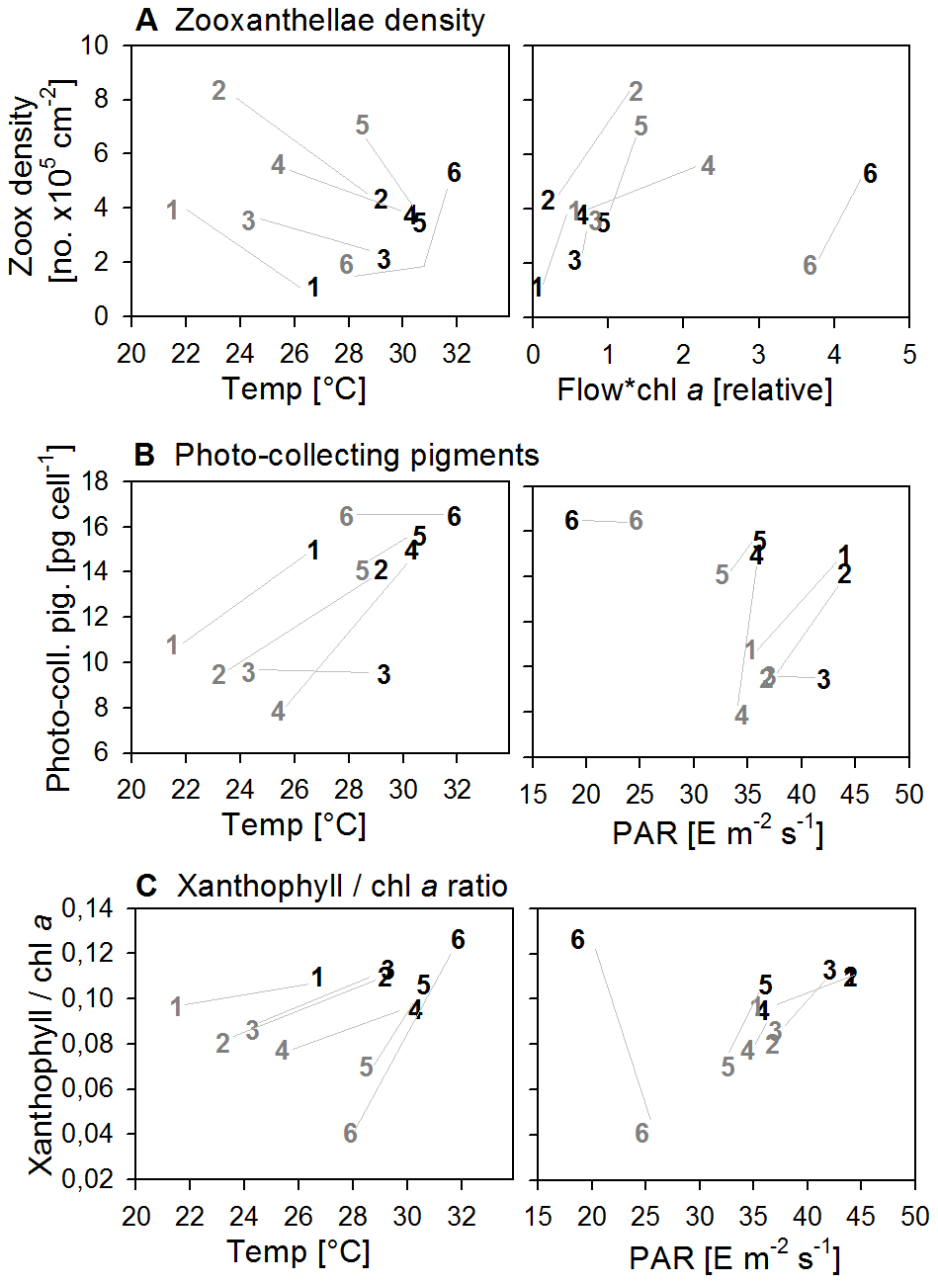
Relationship between environmental parameters and zooxanthellae composition at all sites (1–6) in Sep11 (black) and Mar12 (grey). Data points represent means (standard errors are listed in table S2). The lines connect the two seasons within each site. Light intensity (PAR) in 5 m depth.

We divided zooxanthellae characteristics into cell properties and performance characteristics. The cell properties included zooxanthellae density, concentration and ratios of photo-collecting and photo-protective pigments and concentration of proteins. Most of the parameters showed a latitudinal and/or seasonal pattern, although patterns were usually accompanied by one or two exceptions. Zooxanthellae density increased more than 5-fold from north to south in Sep11, but was exceptionally high at 2-WAJ, while a latitudinal trend was lacking in Mar12 (Fig. 3A). Seasonality was evident at all sites with 2- to 3-times higher zooxanthellae densities in Mar12, except at 6-FAR, where zooxanthellae density decreased (Fig. 3A). The concentration of photo-collective pigments (sum of chl *a*, *c2* and peridinin) per cell lacked a latitudinal trend in both seasons, but was generally highest in the two southern reefs 5-DOG and 6-FAR (Fig. 3B). They decreased 1.5- to 2-fold from Sep11 to Mar12 at 1-MAQ, 2-WAJ and 4-JED, but remained rather stable at the other sites (Fig. 3B). The relative abundance of the photo-protective pigment xanthophyll compared to the major photo-collecting pigment chl *a* (xantho/chl *a*) decreased from north to south in Sep11 by a magnitude of 2.5, but was consistently high in Sep11. Hence, seasonal changes were highest in the south with a 3-fold decrease of xantho/chl *a* from Sep11 to Mar12 and almost absent at 1-MAQ (Fig. 3C). Protein concentrations per cell neither showed a latitudinal nor a seasonal trend and varied between 64.5±3.2 and 109±11 pg cell^−1^ (Table S2).

**Figure 4:**
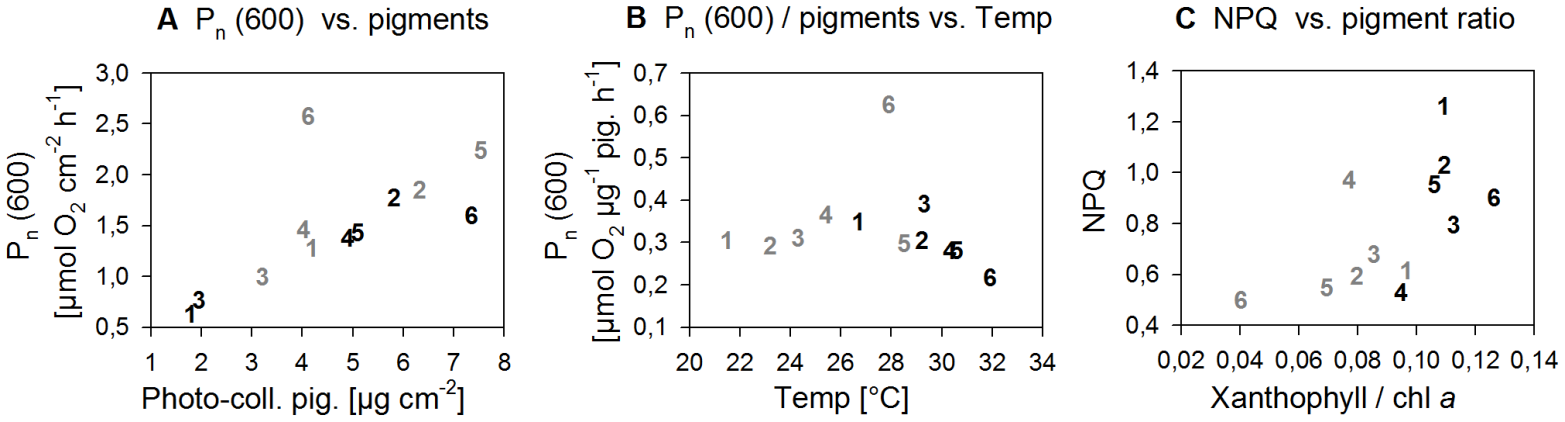
Zooxanthellae performance plotted against A)+C) zooxanthellae composition and B) temperature at all sites (1–6) in Sep11 (black) and Mar12 (grey). Data points represent means (standard errors are listed in table S1 and S2).

Zooxanthellae performance included the following parameters: P_n_(600) standardized to coral surface area, to zooxanthellae number and to µg photo-collecting pigments, F_v_/F_m_ and NPQ (Table S3). Within these parameters, geographic variability occurred, although they did not follow a clear latitudinal trend for the majority of cases. In Mar12, highest areal P_n_(600) rates (photosynthetic output) were found in the southern reefs (5-DOG, 6-FAR) with >2.2 µmol O_2_ cm^−2^ h^−1^, while in Sep11, highest rates occurred at 2-WAJ and 6-FAR with >1.6 µmol O_2_ cm^−2^ h^−1^ (Fig. 4A). Looking at the P_n_ rates per 10^5^ cells and per µg photo-collecting pigments (photosynthetic efficiency) in Sep11, lowest rates were found at 6-FAR with 0.30±0.10 and 0.22±0.06 µmol O_2_ h^−1^, respectively. Therefore, high areal photosynthetic rates at 6-FAR in Sep11 can be ascribed to the high zooxanthellae number. Seasonal trends were more consistent than latitudinal trends, where higher performances were found in Mar12 than in Sep11 concerning areal P_n_(600) (increased <2-fold) and F_v_/F_m_ (increase 1- to 1.2-fold, values between 0.55 and 0.67). However, in contrast, a higher photosynthetic efficiency was found in Sep11, where P_n_(600) per 10^5^ cells was 1.3- to 2-fold higher compared to Mar12 (except at 6-FAR), as well as a higher photoprotective activity, where NPQ values were 1.2- to 2-fold higher in Sep11 than in Mar12 (except at 4-JED). A complete list of parameters, values and associated standard errors of the zooxanthellae characteristics is provided as Supporting Information (Table S2 and S3), as well as the P-I and NPQ-I curves (Fig. S3 and S4).

**Figure 5:**
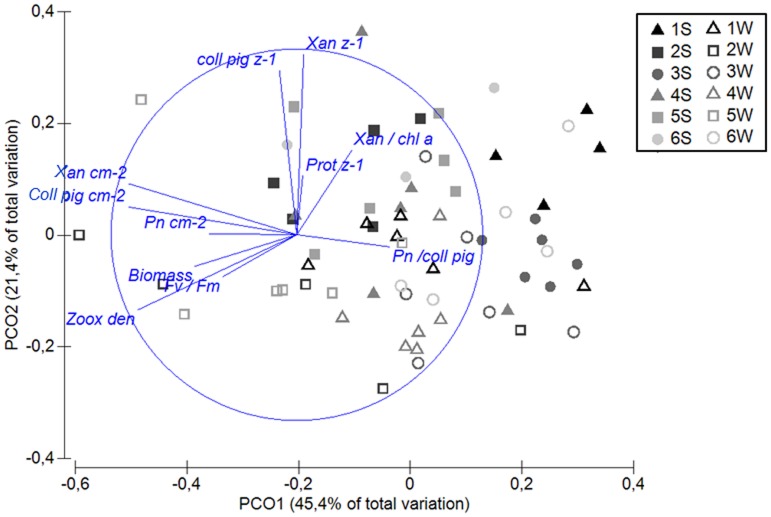
Results of principal coordinate analysis (PCO) (equivalent to PCA, but distances based on chi statistics). Each point represents the combined characteristics of the zooxanthellae at a certain site (1–6) and season (S = end of summer-Sep11, W = end of winter-Mar12) and the vectors represent the importance (length) and direction of the variables in explaining the variation between the points. Only variables contributing to explanation with R^2^>0.4 are indicated. Xan = Xanthophyll, coll pig = photo-collecting pigments, Prot = protein, Zoox den = zooxanthellae density, z^−1^ = per zooxanthellae, Pn = net photosynthesis at PAR 600, NPQ = non-photochemical quenching at PAR 600.

The trends of zooxanthellae characteristics were analyzed by PCO analysis. Here, PCO1 could be mainly associated with geographic variation and PCO2 with seasonal variation (Fig. 5). PCO1 was primarily composed of parameters standardized to surface area (cm^−2^), i.e. zooxanthellae density, protein, photo-collecting pigments, as well as P_n_(600). However, although these parameters varied greatly between sites, they could not be associated to a clear north-south trend (as indicated by the color intensity in Fig. 5), as partly described above. Seasonal changes manifested mainly on a cellular level, as indicated by enhanced pigmentation per zooxanthellae cell in Sep11 (PCO2), which was most pronounced for xanthophyll, but also for the photo-collecting pigments and xantho/chl *a* ratio (Fig. 5). Although we found differences in regard to geographic and seasonal variations, these were not strongly correlated with dominant *Symbiodinium* composition. The relationship between *Symbiodinium* composition and physiological characteristics was weak. Clade A explained 5.1% and clade C explained 4.5%, while clade D did not contribute to explained variation in zooxanthellae characteristics (Table 1A).

**Table 1:** Relationship between zooxanthellae characteristic (response variable – resemblance matrix) and **A**) clade composition (predictor variable – resemblance matrix), and **B**) environmental parameter (predictor variable) (DISTLM results).

A				
Pred. var.	SS	Pseudo-F	P	Probability [%]
A	0.224	3.21	**0.007**	5.1
C	0.196	2.80	**0.020**	4.5

B				
Pred. var.	SS	Pseudo-F	P	Probability [%]
Temp	0.405	5.954	**0.001**	8.8
Flow	0.337	4.899	**0.001**	7.3
Flow*chl *a*	0.210	2.969	**0.016**	4.6

Significant results in bold.

### Relationship between environment and *Symbiodinium* characteristics

Looking at *Symbiodinium* characteristics in total (distance based resemblance matrix), the most explaining environmental parameter was found to be temperature with 8.8% explained variation, followed by flow (7.3%), and nutrient flux (4.6%) (Table 1B).

**Figure 6:**
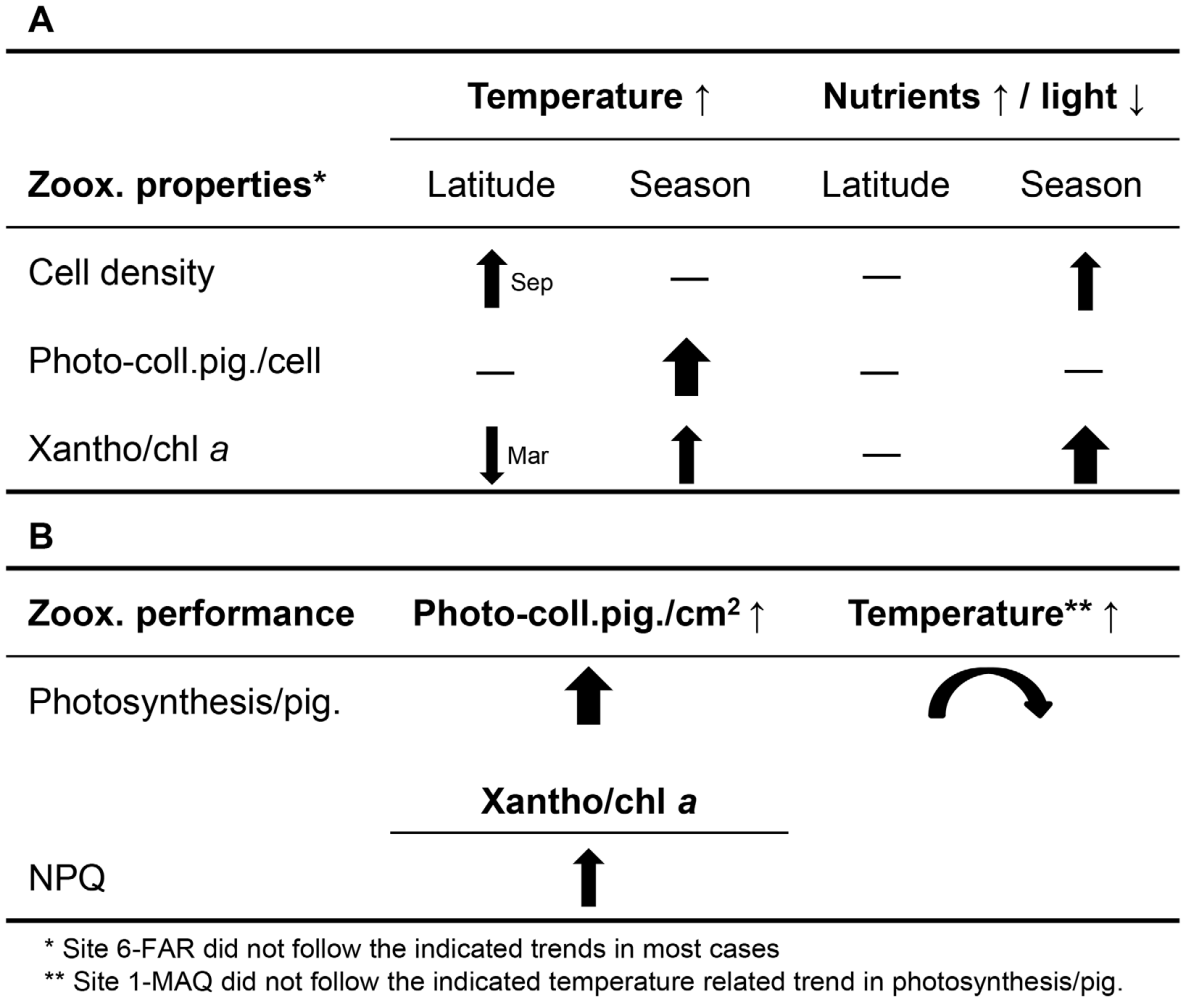
Summary of results. **A**) Relationships found between the environmental parameters (temperature and nutrients/light) and the zooxanthellae (zoox.) properties (density, photo-collecting pigments/cell and xantho/chl *a* ratio [relative abundance of photo-protective pigments]) over the latitudes and between seasons. The addition Sep or Mar next to the arrow indicates the months of the observed latitudinal trend. **B**) Relationships found between zooxanthellae properties and zooxanthellae performance and between zooxanthellae performance (here only photosynthesis standardized to photo-collecting pigments [photosynthesis/pig.]) and temperature. The directions of the arrows indicate an increase (upward) or decrease (downward) of the corresponding zooxanthellae property or performance, the thickness indicates the relative strength of relationship.

Looking at individual zooxanthellae properties the following patterns were found (summarized in Fig. 6), based on partial correlation and multiple regression (Table S4). On a spatial scale, zooxanthellae density was positively related to temperature in Sep11 (but not in Mar12), but not to nutrients in any of the seasons (Fig. 3A, Fig. 6). However, on a temporal scale, zooxanthellae density increased with nutrient supply from Sep11 to Mar12, if the exceptional site 6-FAR is ignored (Fig. 6). Here, highest zooxanthellae density coincided with the most nutrient enriched water in Sep11 (Fig. 3A). The concentration of photo-collecting pigments per cell significantly increased with temperature, regarding seasonal changes (Fig. 3B, Fig. 6). In contrast, light intensity at 5 m depth seemed to be more related to geographic differences, although, but this effect was almost exclusively driven by 6-FAR, where high pigment concentration coincided with low light intensity (Fig. 3B, Fig. 6). The xantho/chl *a* ratio significantly increased with increasing light intensity and temperature (Fig. 3C), if the environmental parameters were considered separately (partial correlation), as well as together (stepwise forward regression) (Table S4), except at 6-FAR. While light effects could mainly be related to seasonal changes in pigment composition, temperature could also be related to geographic variability, although in a contradictory way (Fig. 3C, Fig. 6). Here, a negative relationship between temperature and xantho/chl *a* ratio was found in Mar12. Interestingly, seasonal effects became stronger towards the south, despite a decrease in seasonal variation of temperature (Fig. 3C).

Concerning zooxanthellae performance, areal P_n_(600) was a linear function of areal concentration of photo-collecting pigments, except at site 6-FAR (Fig. 4A) (all sites: r^2^ = 0.17, p<0.05; sites 1–5: r^2^ = 0.40, p<0.05). However, photosynthetic efficiency (here, photosynthesis standardization to photo-collecting pigments) also seemed to be related to temperature, revealing a slight trend towards an optimum between 26 and 28°C (Fig. 4B). Only 1-MAQ maintained photosynthetic efficiency on a relatively high level at cold temperatures (Fig. 4B). NPQ(600) was positively related to the xantho/chl *a* ratio (Fig. 4C) (r^2^ = 0.30, p<0.05), however NPQ(600) could not be related to temperature directly.

## Discussion

Despite pronounced differences in prevailing environmental conditions (i.e. temperature and nutrient input) from the northern to the southern Red Sea, this study revealed a surprisingly low latitudinal differentiation in *Symbiodinium* type and physiology of the coral *P. verrucosa*. Only the northernmost reef, in the Gulf of Aqaba, was distinct in regard to dominant symbiont type and partly in its physiology, indicating that the Gulf of Aqaba forms a discrete habitat from the Red Sea proper. In contrast to the absence of strong latitudinal trends, the seasonal trend in *Symbiodinium* physiology was much more pronounced. This is, although environmental parameters generally changed stronger with latitudes than with seasons. In particular, nutrient supply increased strongly from north to south, while seasonal variation decreased (latitudinal variation in summer: >70-fold, in winter: 6.6-fold, seasonal fluctuation in the north: 9.2-fold, in the S: 1.2-fold). Also temperature showed a larger seasonal variation in the north (21–27°C) compared to the south (28–32/33°C). Further, light availability (up to 2-fold different) correlated negatively with nutrient availability, due to a nutrient-driven increase in turbidity [16]. In the following, we provide a detailed discussion on spatial patterns of dominant *Symbiodinium* types and their physiological characteristics, followed by a discussion on seasonal differences of *Symbiodinium* physiology.

### Spatial patterns of *Symbiodinium* abundance and physiology

The two dominant *Symbiodinium* clades identified in our study, type A1 (*S. microadriaticum*) and clade C, were previously found in the Red Sea proper and the Gulf of Aqaba [26], [54]. Our data indicate a change in the dominant clade from clade A to C between the Red Sea proper and the Gulf of Aqaba. Although the sample size of this study was relatively low, our data was corroborated by a recent study of the same authors (unpublished data). Here, an extensive evaluation of the clades associated with *P. verrucosa* throughout the Saudi-Arabian Red Sea was carried out on a set of 278 samples, covering the same sampling sites of the study. In these data, it was found that 85% of *P. verrucosa* was dominated by ITS2 type A1 symbionts, while 10% were dominated by clade C (mainly type C1 – *S. goreaui*). Clade C was found almost exclusively in the northern regions (1-MAQ and less at 2-WAJ) and only 5% of the samples were associated with other clades, including ITS2 types from clades B and D or a combination of A and C. Clade C was therefore dominant only in the nutrient-depleted and coldest region of the Red Sea. Similarly, type C1 was also dominant in the congener *Pocillopora damicornis* in the Great Barrier Reef, where the temperature regime (20.5°C and 29.5°C) was similar to the Gulf of Aqaba [55], inferring a certain ‘cold’-water preference of this clade. The photosynthetic efficiency (P_n_ standardized to photo-collecting pigments) was comparatively high in corals dominated by clade C despite low temperatures, and NPQ was strongly regulated despite only a small change in the xantho/chl *a* ratio. Being aware that differences in NPQ may partly also be attributed to differences in the degree to which the photosystem is adjusted to the current light intensity (full adjustment never reached during rapid light curves [56]), our results demonstrate a comparatively high *Symbiodinium* performance of clade C dominated coral communities. This may be necessary to counterbalance the particularly low cell density in these communities (at 1-MAQ).

In contrast to the Gulf of Aqaba, however, the Red Sea proper was consistently dominated by *S. microadriaticum* covering a temperature range of 23°C to 33°C. Species of *Symbiodinium* clade A were previously found to be particularly thermo-tolerant due to their high capability to adjust their photo-protective pathways [57]. This is supported by our results, where both, xantho/chl *a* ratios and NPQ, increased with increasing temperature and light from winter to summer. This seasonal adjustment was particularly prominent in the most southern reef Farasan, where highest temperatures occurred during summer. Here we also identified a distinct ITS2 type (i.e. A21) that runs close to the DGGE fingerprint of type A1. Future studies should address the ecological significance of this difference. Light in contrast to temperature, however, seemed to have a lower impact on the photo-protective mechanism (at least in the southern Red Sea), although both parameters, are known to enhance xanthophyll abundance and cycling (reviewed [4]). Our results indicate that *S. microadriaticum* is particularly capable to adjust to a broad temperature regime and to temperature extremes (>31°C) by a high capacity to adjust its photo-protective mechanism.

The high ITS2 type fidelity throughout the Red Sea proper is consistent with previous studies on pocilloporid corals, for example along the latitudinal environmental gradient of the Great Barrier Reef [55], after vertical transplantation of corals in the Eastern Pacific [58] and after a cold-water bleaching event in the Gulf of Mexico [59]. However, it needs to be emphasized that in the Red Sea, high clade fidelity occurred despite temperature extremes in the South, which infers either long-term acclimatization or genetic differentiation of *S. microadriaticum* to local conditions.

Although it remains highly speculative, the divergence of the rDNA of ITS2 type A1 from the most southern reef Farasan (A21) compared to the other reefs may hint towards a possible adaptation to environmental conditions in the south. The largely exceptional behavior of the zooxanthellae at Farasan may further support this hypothesis. However, another reason for the exceptional behavior of zooxanthellae at Farasan may be found in a non-linear response to changes in environmental conditions. For example nutrient flux was 2- to 20-fold higher at Farasan compared to the other regions, while zooxanthellae density remained within the range of the other regions, indicating a limited capacity for zooxanthellae proliferation or a higher throughput of zooxanthellae (high division and expulsion rates).

Nutrients, however, seemed to have a positive effect on zooxanthellae density in summer when densities increased from north to south. Nutrients thereby may have had a counterbalancing effect on high summer temperatures (in the central and southern reefs), which are most likely responsible for the observed decrease in photosynthetic efficiency (P_n_ standardized to photo-collecting pigments) from north to south. The thereby obtained constant areal photosynthetic rates during summer in the central and southern reefs, ensuring high gain of photosynthetic energy, leads us to the conclusion that increased nutrient input in the south is rather beneficial than detrimental for the coral *P. verrucosa* to withstand high temperatures.

### Seasonal patterns of *Symbiodinium* physiology

Seasonal patterns were observed in all measured parameters concerning zooxanthellae physiology. While zooxanthellae densities and xantho/chl *a* ratios were stronger related to nutrient and/or light changes, photo-collecting pigments per cell and photosynthetic efficiency were stronger related to temperature changes.

The lower zooxanthellae density in summer is consistent with our expectation, since it is known to decrease in consequence of nutrient-depletion, high light and high temperature (reviewed [60], [61]). Also the relative increase of photo-protective pigments (xantho/chl *a* ratio) in summer in consequence of increased light (and temperature, as discussed already) is normal [4]. However, the general increase of photo-collecting pigments per cell in summer is contradictory to our expectations. Photo-collecting pigments per cell usually increase with decreasing light to enhance light harvesting capacity [18], [62], [63] and/or they are fostered by increasing nutrient availability [11], [13]. Only Farasan showed a rather common behavior, where high pigmentation occurred in both seasons and coincided with all-year reduced light availability and increased nutrients. An explanation for higher cellular photo-collecting pigments in summer is not trivial. A possible explanation might be, that the minimum of available nutrients in summer are used to enhance the interior of zooxanthellae (increased pigmentation per cell), which might partly counterbalance the loss of zooxanthellae, as supported by our higher photosynthetic rates per cell number in Sep11 (except at 6-FAR). However, it does not seem to be enough to fully counterbalance negative effects of high summer temperatures on the performance of zooxanthellae, as indicated by an overall lower photosynthetic output per coral surface area and lower maximum photosynthetic yields (F_v_/F_m_) in summer, particularly in the central and southern Red Sea. Although the abundance of dominant zooxanthellae clades revealed some differences between summer and winter, more comprehensive sampling is needed to unequivocally resolve whether seasonal shifts in clade community structures are ecologically significant.

In summary, strong host fidelity of *S. microadriaticum* (ITS2 type A1) with its host *P. verrucosa* throughout the Red Sea proper indicates that this coral species displays a wide range of physiological adjustments even to extreme environmental conditions that persist in this region. Relatively greater temporal, as opposed to spatial variations in zooxanthellae physiology, despite the high variation of environmental conditions on both scales, indicates long-term acclimatization or adaptations to regional conditions of *S. microadriaticum*. Adaptation may have occurred in the most southern region, where a divergent ITS2 type to A1 (A21) was found. Compensatory effects of nutrients in the high temperature southern region seem to be the case, where increased zooxanthellae density compensates for a loss of photosynthetic performance, although this needs further investigations for confirmation. The dominance of clade C in the Gulf of Aqaba, assigns this common clade, or at least the identified types, to a less high temperature tolerant clade, while it seems to be a better performer in very low nutrient environments.

While this study is the first to provide important insights into the complexity of coral symbiont responses along the latitudinal gradient of the Red Sea, future studies on population genetics and controlled laboratory studies with corals from the different regions will help to differentiate more clearly between acclimatization versus adaptation processes. Also, studies on the coral host (as well as associated bacterial communities) are likely to increase our understanding about mechanisms of holobiont resilience and the modest trend in physiological differences in *Symbiodinium* in relation to latitudinal differences.

## Supporting Information

Figure S1 **Image of incubation chambers.** Four acrylic chambers with a volume of 950 ml, each equipped with a battery-run stirrer, a water inlet and outlet with one-way valves just before and after the incubation chambers respectively, a fragment holder and an oxygen sensor (DIGISENS-Optod, Ponsel, France.). The oxygen sensors were connected to a battery-run data logger (SDL500 Submersible Data Logger, NexSens Technology, USA). An UW-PAR sensor (Li-192, LiCor Biosciences GmbH, Germany) was installed at the height of the chambers and connected to the same data logger. Each water inlet was connected to a central programmable battery-run pump via tubing, which pumped the surrounding water through the chambers at a given interval and duration. All components of the setup were fixed to a POM (Polyoxymethylen) frame; the data logger and pump were fixed below the chambers.
(DOCX)

Figure S2 **Photographs of 3 representative DGGE gels showing the prominent bands with the corresponding clade names.**
(PDF)

Figure S3 **P-I curves at all sites from N (top) to S (bottom) in September 2011 and March 2012.** Mean± SE.
(DOCX)

Figure S4 **NPQ versus PAR at all sites from N (top) to S (bottom) in September 2011 and March 2012.** Mean± SE.
(DOCX)

Table S1 **NASA-derived environmental data at all sites (1–6, North – South) in September 2011(Sep11) and March 2012 (Mar12).** Detailed description of data is provided within the table.
(XLSX)

Table S2 **Zooxanthellae properties at all sites (1–6, North – South) in September 2011(Sep11) and March 2012 (Mar12).** Photo-collecting pigments: sum of chlorophyll *a* and *c2* and peridinin. N = 6. Mean (±SE).
(DOCX)

Table S3 **Zooxanthellae performance at all sites (1–6, North – South) in September 2011(Sep11) and March 2012 (Mar12).** Net photosynthesis and non-photochemical quenching (NPQ) at PAR 600 µmol photons m^−2^ s^−1^ (600), photo-collecting pigments (photo-coll. pig.). Maximum photosynthetic yield (F_v_/F_m_). N = 6. Mean (±SE).
(DOCX)

Table S4 **Results of correlation and regression analyses.** Dependent variables are the zooxanthellae characteristics density, photo-collecting pigments, and protein cell^−1^ and the ratio xanthophyll/chl *a*. The predictor variables (pred. var.) include the environmental parameters temperature (**T**), nutrients (**N** [flow*chl *a*]) and light (**L** [PAR]). Significant results are in bold (p>0.05). Analyses were conducted with complete data sets (season: all, site: all) and seasonal sub-sets (season: Sep or Mar, site: all) to differentiate between overall patterns across sites and seasons and pure geographic patterns (N-S), respectively. Furthermore, comparison of results between complete data sets and seasonal sub-sets allowed discriminating between seasonal and geographic responses to environmental variability. Since the correlation between temperature and nutrients or light was always significant within the seasonal sub-sets, no stepwise forward regression analyses were performed with these data sets, but partial correlation only.
(DOCX)

## References

[1] Jones RJ, Hoegh-Guldberg O, Larkum AWD, Schreiber U (1998) Temperature-induced bleaching of corals begins with impairment of the CO2 fixation mechanism in zooxanthellae. Plant Cell and Environment 21: 1219–1230.

[2] Glynn PW (1996) Coral reef bleaching: facts, hypotheses and implications. Global Change Biology 2: 495–509.

[3] Grottoli AG, Rodrigues LJ, Palardy JE (2006) Heterotrophic plasticity and resilience in bleached corals. Nature 440: 1186–1189. 16641995 10.1038/nature04565

[4] Coles SL, Brown BE (2003) Coral bleaching — capacity for acclimatization and adaptation. Advances in Marine Biology: Academic Press. pp. 183–223. 10.1016/s0065-2881(03)46004-5 14601413

[5] Weis VM (2008) Cellular mechanisms of Cnidarian bleaching: stress causes the collapse of symbiosis. Journal of Experimental Biology 211: 3059–3066. 18805804 10.1242/jeb.009597

[6] Sheppard CRC, Sheppard ALS (1991) Corals and coral communities of Arabia. Fauna of Saudi Arabia 12: 3–170.

[7] Sawall Y, Al-Sofyani A, Kürten B, Al-Aidaroos AM, Hoang BX, et al (2014) Coral communities, in contrast to fish communities, maintain a high assembly similarity along the large latitudinal gradient along the Saudi Red Sea coast. Journal of Ecology and Ecography S4: 003 doi: 10.4172/2157-7625.S4-003. In press

[8] Stott PA, Tett SFB, Jones GS, Allen MR, Mitchell JFB, et al (2000) External Control of 20th Century Temperature by Natural and Anthropogenic Forcings. Science 290: 2133–2137. 11118145 10.1126/science.290.5499.2133

[9] Hoegh-Guldberg O (1999) Climate change, coral bleaching and the future of the world's coral reefs. Marine and Freshwater Research 50: 839–866.

[10] Rabalais NN, Turner RE, Díaz RJ, Justić D (2009) Global change and eutrophication of coastal waters. ICES Journal of Marine Science: Journal du Conseil 66: 1528–1537.

[11] Dubinsky Z, Stambler N, Ben-Zion M, Mccloskey LR, Muscatine L, et al (1990) The Effect of External Nutrient Resources on the Optical Properties and Photosynthetic Efficiency of Stylophora pistillata. Proceedings of the Royal Society of London B Biological Sciences 239: 231–246.

[12] Houlbrèque F, Ferrier-Pagès C (2008) Heterotrophy in Tropical Scleractinian Corals. Biological Reviews 84: 1–17. 19046402 10.1111/j.1469-185X.2008.00058.x

[13] Sawall Y, Teichberg MC, Seemann J, Litaay M, Jompa J, et al (2011) Nutritional status and metabolism of the coral *Stylophora subseriata* along a eutrophication gradient in Spermonde Archipelago (Indonesia). Coral Reefs 30: 841–853.

[14] Dubinsky Z, Jokiel PL (1994) Ratio of energy and nutrient fluxes regulates symbiosis between zooxanthellae and corals. Pacific Science 48: 313–324.

[15] Wiedenmann J, D'Angelo C, Smith EG, Hunt AN, Legiret F-E, et al (2013) Nutrient enrichment can increase the susceptibility of reef corals to bleaching. Nature Climate Change 3: 160–164.

[16] Fabricius KE (2005) Effects of terrestrial runoff on the ecology of corals and coral reefs: review and synthesis. Marine Pollution Bulletin 50: 125–146. 15737355 10.1016/j.marpolbul.2004.11.028

[17] Titlyanov EA, Titlyanov TV, Yamazato K, van Woesik R (2001) Photo-acclimation of the hermatypic coral Stylophora pistillata while subjected to either starvation or food provisioning. Journal of Experimental Marine Biology and Ecology 257: 163–181. 11245874 10.1016/s0022-0981(00)00308-7

[18] Mass T, Einbinder S, Brokovich E, Shashar N, Vago R, et al (2007) Photoacclimation of Stylophora pistillata to light extremes: metabolism and calcification. Marine Ecology-Progress Series 334: 93–102.

[19] Brown BE, Downs CA, Dunne RP, Gibb SW (2002) Exploring the basis of thermotolerance in the reef coral *Goniastrea aspera* . Marine Ecology Progress Series 242: 119–129.

[20] Ambarsari I, Brown BE, Barlow RG, Britton G, Cummings D (1997) Fluctuations in algal chlorophyll and carotenoid pigments during solar bleaching in the coral Goniastrea aspera at Phuket, Thailand. Marine Ecology Progress Series 159: 303–307.

[21] Robison JD, Warner ME (2006) Differential impacts of photoacclimation and thermal stress on the photobiology of four different phylotypes of *Symbiodinium* (Pyrrhophyta). Journal of Phycology 42: 568–579.

[22] Frade PR, De Jongh F, Vermeulen F, Van Bleijswijk J, Bak RPM (2008) Variation in symbiont distribution between closely related coral species over large depth ranges. Molecular Ecology 17: 691–703. 18179427 10.1111/j.1365-294X.2007.03612.x

[23] LaJeunesse TC, Pettay DT, Sampayo EM, Phongsuwan N, Brown B, et al (2010) Long-standing environmental conditions, geographic isolation and host-symbiont specificity influence the relative ecological dominance and genetic diversification of coral endosymbionts in the genus Symbiodinium. Journal of Biogeography 37: 785–800.

[24] Fabricius KE, Mieog JC, Colin PL, Idip D, Van Oppen MJ (2004) Identity and diversity of coral endosymbionts (zooxanthellae) from three Palauan reefs with contrasting bleaching, temperature and shading histories. Molecular Ecology 13: 2445–2458. 15245416 10.1111/j.1365-294X.2004.02230.x

[25] Berkelmans R, van Oppen MJH (2006) The role of zooxanthellae in the thermal tolerance of corals: a ‘nugget of hope’ for coral reefs in an era of climate change. Proceedings of the Royal Society B: Biological Sciences 273: 2305–2312. 16928632 10.1098/rspb.2006.3567 PMC1636081

[26] Baker AC, Starger CJ, McClanahan TR, Glynn PW (2004) Coral reefs: Corals' adaptive response to climate change. Nature 430: 741–741. 15306799 10.1038/430741a

[27] Goulet TL (2006) Most corals may not change their symbionts. Marine Ecology Progress Series 321: 1–7.

[28] Jessen C, Villa L, Javier F, Bayer T, Roder C, et al (2013) *In-situ* effects of eutrophication and overfishing on physiology and bacterial diversity of the Red Sea Coral *Acropora hemprichii* . PLoS ONE 8: e62091. 23630625 10.1371/journal.pone.0062091 PMC3632597

[29] Borell EM, Bischof K (2008) Feeding sustains photosynthetic quantum yield of a scleractinian coral during thermal stress. Oecologia 157: 593–601. 18618148 10.1007/s00442-008-1102-2

[30] Ferrier-Pagès C, Rottier C, Beraud E, Levy O (2010) Experimental assessment of the feeding effort of three scleractinian coral species during a thermal stress: Effect on the rates of photosynthesis. Journal of Experimental Marine Biology and Ecology 390: 118–124.

[31] Stimson J, Sakai K, Sembali H (2002) Interspecific comparison of the symbiotic relationship in corals with high and low rates of bleaching-induced mortality. Coral Reefs 21: 409–421.

[32] Souvermezoglou E, Metzl N, Poisson A (1989) Red Sea budgets of salinity, nutrients and carbon calculated in the Strait of Bab-El-Mandab during the summer and winter seasons. Journal of Marine Research 47: 441–456.

[33] Kleypas JA, McManus JW, Menez LAB (1999) Environmental limits to coral reef development: Where do we draw the line? American Zoologist 39: 146–159.

[34] Bell PRF (1992) Eutrophication and coral reefs–some examples in the Great Barrier Reef lagoon. Water Research 26: 553–568.

[35] Veron JEN, editor (2000) Corals of the world: Australian Institute of Marine science and CRR Qld Pty Ltd.

[36] DeVantier L, Turak E, Al-Shaikh K (2000) Coral bleaching in the central-northern Saudi Arabian Red Sea, August–September 1998. In: Tatwany H, editor; Riyadh, Saudi Arabia. National Commission for Wildlife Conservation and Development.

[37] Jokiel PL, Morrissey JI (1993) Water Motion on Coral Reefs - Evaluation of the Clod Card Technique. Marine Ecology Progress Series 93: 175–181.

[38] Acker J, Leptoukh G, Shen S, Zhu T, Kempler S (2008) Remotely-sensed chlorophyll a observations of the northern Red Sea indicate seasonal variability and influence of coastal reefs. Journal of Marine Systems 69: 191–204.

[39] Loisel H, Nicolas J-M, Sciandra A, Stramski D, Poteau A (2006) Spectral dependency of optical backscattering by marine particles from satellite remote sensing of the global ocean. Journal of Geophysical Research: Oceans 111: C09024.

[40] Dennison WC, Orth RJ, Moore KA, Stevenson JC, Carter V, et al (1993) Assessing Water Quality with Submersed Aquatic Vegetation. BioScience 43: 86–94.

[41] Ralph PJ, Gademann R (2005) Rapid light curves: A powerful tool to assess photosynthetic activity. Aquatic Botany 82: 222–237.

[42] Maxwell K, Johnson GN (2000) Chlorophyll fluorescence - a practical guide. Journal of Experimental Botany 51: 659–668. 10938857 10.1093/jxb/51.345.659

[43] Naumann MS, Niggl W, Laforsch C, Glaser C, Wild C (2009) Coral surface area quantification-evaluation of established techniques by comparison with computer tomography. Coral Reefs 28: 109–117.

[44] LaJeunesse TC, Trench RK (2000) Biogeography of two species of *Symbiodinium* (Freudenthal) inhabiting the intertidal sea anemone *Anthopleura elegantissima* (Brandt). Biological Bulletin 199: 126–134. 11081711 10.2307/1542872

[45] LaJeunesse TC, Loh WKW, van Woesik R, Hoegh-Guldberg O, Schmidt GW, et al (2003) Low symbiont diversity in southern Great Barrier Reef corals, relative to those of the Caribbean. Limnology and Oceanography 48: 2046–2054.

[46] Sampayo EM, Dove S, Lajeunesse TC (2009) Cohesive molecular genetic data delineate species diversity in the dinoflagellate genus *Symbiodinium* . Molecular Ecology 18: 500–519. 19161470 10.1111/j.1365-294X.2008.04037.x

[47] LaJeunesse TC (2002) Diversity and community structure of symbiotic dinoflagellates from Caribbean coral reefs. Marine Biology 141: 387–400.

[48] Tamura K, Peterson D, Peterson N, Stecher G, Nei M, et al (2011) MEGA5: Molecular Evolutionary Genetics Analysis Using Maximum Likelihood, Evolutionary Distance, and Maximum Parsimony Methods. Molecular Biology and Evolution 28: 2731–2739. 21546353 10.1093/molbev/msr121 PMC3203626

[49] Edgar RC (2004) MUSCLE: multiple sequence alignment with high accuracy and high throughput. Nucleic Acids Research 32: 1792–1797. 15034147 10.1093/nar/gkh340 PMC390337

[50] Franklin EC, Stat M, Pochon X, Putnam HM, Gates RD (2012) GeoSymbio: a hybrid, cloud-based web application of global geospatial bioinformatics and ecoinformatics for *Symbiodinium*-host symbiosis. Molecular Ecology Resources 12: 369–373. 22018222 10.1111/j.1755-0998.2011.03081.x

[51] Anderson MJ, Gorley RN, Clarke KR (2008) PERMANOVA+ for PRIMER: Guide to software and statistica methods. Plymouth, United Kingdom: PRIMER-E Ltd.

[52] Trench RK, Blank RJ (1987) *Symbiodinium microadriaticum* Freudenthal, *S. goreauii* sp. nov., *S. kawagutii* sp. nov. and *S. pilosum* sp. nov.: Gymnodinioid dinoflagellate symbionts of marine invertebrates. Journal of Phycology 23: 469–481.

[53] LaJeunesse TC (2001) Investigating the biodiversity, ecology, and phylogeny of endosymbiotic dinoflagelltes in the genus *Symbiodinium* using the ITS region: In search of a “species” level marker. Journal of Phycology 37: 866–880.

[54] Karako-Lampert S, Katcoff DJ, Achituv Y, Dubinsky Z, Stambler N (2004) Do clades of symbiotic dinoflagellates in scleractinian corals of the Gulf of Eilat (Red Sea) differ from those of other coral reefs? Journal of Experimental Marine Biology and Ecology 311: 301–314.

[55] Ulstrup KE, Berkelmans R, Ralph PJ, van Oppen MJH (2006) Variation in bleaching sensitivity of two coral species across a latitudinal gradient on the Great Barrier Reef: the role of zooxanthellae. Marine Ecology Progress Series 314: 135–148.

[56] Enríquez S, Borowitzka M (2010) The Use of the Fluorescence Signal in Studies of Seagrasses and Macroalgae. In: Suggett DJ, Prášil O, Borowitzka MA, editors. Chlorophyll a Fluorescence in Aquatic Sciences: Methods and Applications: Springer Netherlands. pp. 187–208.

[57] Reynolds JM, Bruns BU, Fitt WK, Schmidt GW (2008) Enhanced photoprotection pathways in symbiotic dinoflagellates of shallow-water corals and other cnidarians. Proceedings of the National Academy of Sciences 105: 13674–13678. 10.1073/pnas.0805187105 PMC2527352 18757737

[58] Iglesias-Prieto R, Beltrán VH, LaJeunesse TC, Reyes-Bonilla H, Thomé PE (2004) Different algal symbionts explain the vertical distribution of dominant reef corals in the eastern Pacific. Proceedings of the Royal Society B: Biological Sciences 271: 1757–1763. 15306298 10.1098/rspb.2004.2757 PMC1691786

[59] McGinley MP, Aschaffenburg MD, Pettay DT, Smith RT, LaJeunesse TC, et al (2012) Symbiodinium spp. in colonies of eastern Pacific Pocillopora spp. are highly stable despite the prevalence of low-abundance background populations. Marine Ecology Progress Series 462: 1–7.

[60] Fitt W, Brown B, Warner M, Dunne R (2001) Coral bleaching: interpretation of thermal tolerance limits and thermal thresholds in tropical corals. Coral Reefs 20: 51–65.

[61] Al-Sofyani A, Floos YAM (2013) Effect of temperature on two reef-building corals *Pocillopora damicornis* and *P. verrucosa* in the Red Sea. Oceanologia 55: 917–935.

[62] Brown BE, Dunne RP, Ambarsari I, Le Tissier MDA, Satapoomin U (1999) Seasonal fluctuations in environmental factors and variations in symbiotic algae and chlorophyll pigments in four Indo-Pacific coral species. Marine Ecology Progress Series 191: 53–69.

[63] Porter JW, Muscatine L, Dubinsky Z, Falkowski PG (1984) Primary production and photoadaptation in light- and shade-adapted colonies of the symbiotic coral, *Stylophora pistillata* . Proceedings of the Royal Society of London Series B, Biological Sciences 222: 161–180.

