# Supplementary material for: Spatio-Temporal Analyses of Symbiodinium Physiology of the Coral Pocillopora verrucosa along Large-Scale Nutrient and Temperature Gradients in the Red Sea
Source: PLoS One. 2014 Aug 19;9(8):e103179. doi: 10.1371/journal.pone.0103179 (PMC4138093; doi:10.1371/journal.pone.0103179)
Supplement: Figure S1 — Image of incubation chambers. Four acrylic chambers with a volume of 950 ml, each equipped with a battery-run stirrer, a water inlet and outlet with one-way valves just before and after the incubation chambers respectively, a fragment holder and an oxygen sensor (DIGISENS-Optod, Ponsel, France.). The oxygen sensors were connected to a battery-run data logger (SDL500 Submersible Data Logger, NexSens Technology, USA). An UW-PAR sensor (Li-192, LiCor Biosciences GmbH, Germany) was installed at the height of the chambers and connected to the same data logger. Each water inlet was connected to a central programmable battery-run pump via tubing, which pumped the surrounding water through the chambers at a given interval and duration. All components of the setup were fixed to a POM (Polyoxymethylen) frame; the data logger and pump were fixed below the chambers. (DOCX) [file pone.0103179.s001.docx]

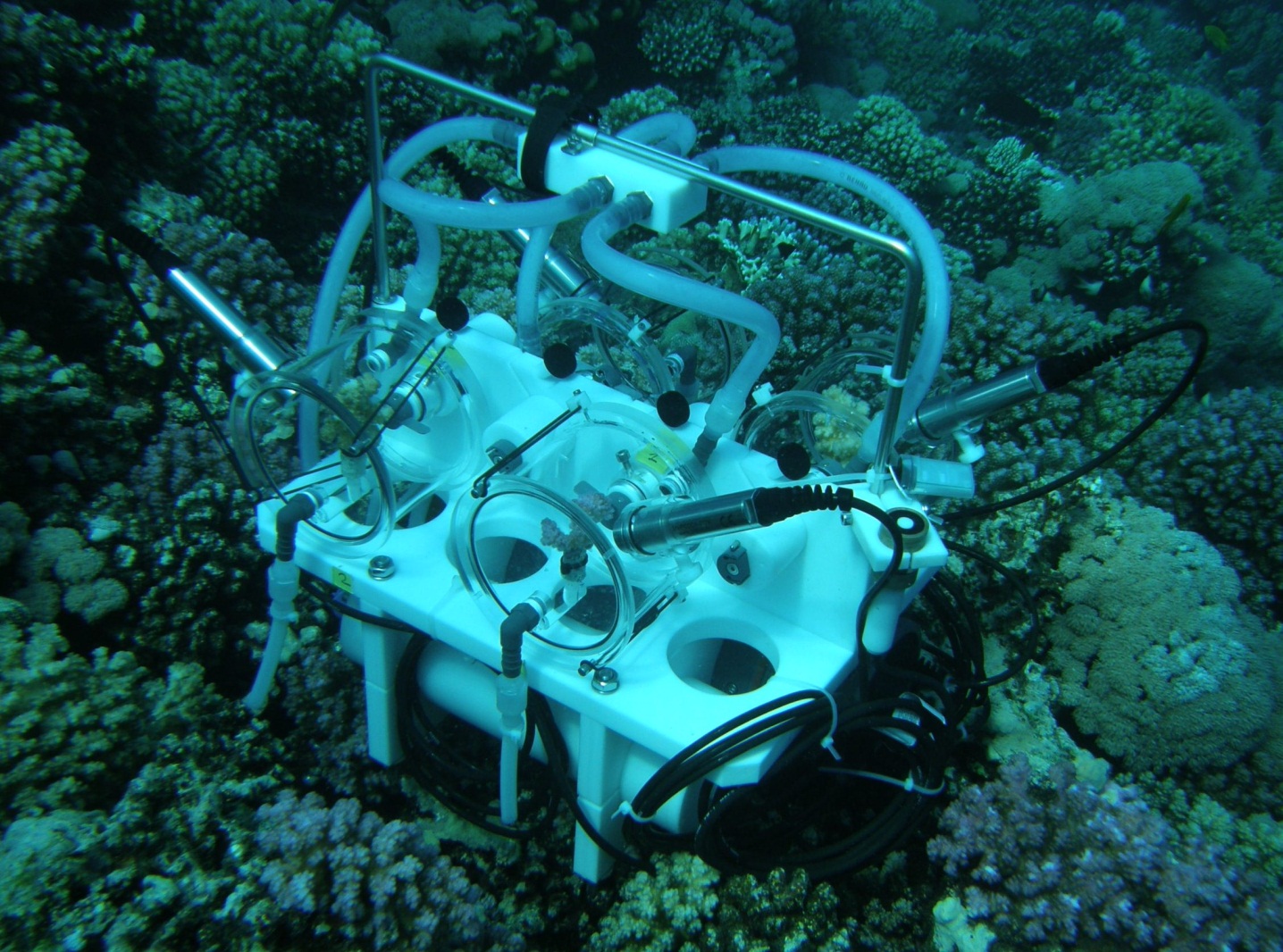


Figure S1. **Photography of incubation chambers.** Four acrylic chambers with a volume of 950 ml, each equipped with a battery-run stirrer, a water inlet and outlet with one-way valves just before and after the incubation chambers respectively, a fragment holder and an oxygen sensor (DIGISENS-Optod, Ponsel, France.). The oxygen sensors were connected to a battery-run data logger (SDL500 Submersible Data Logger, NexSens Technology, USA). An UW-PAR sensor (Li-192, LiCor Biosciences GmbH, Germany) was installed at the height of the chambers and connected to the same data logger. Each water inlet was connected to a central programmable battery-run pump via tubing, which pumped the surrounding water through the chambers at a given interval and duration. All components of the setup were fixed to a POM (Polyoxymethylen) frame; the data logger and pump were fixed below the chambers.
