# Supplementary material for: Spatio-Temporal Analyses of Symbiodinium Physiology of the Coral Pocillopora verrucosa along Large-Scale Nutrient and Temperature Gradients in the Red Sea
Source: PLoS One. 2014 Aug 19;9(8):e103179. doi: 10.1371/journal.pone.0103179 (PMC4138093; doi:10.1371/journal.pone.0103179)
Supplement: Figure S2 — Photographs of 3 representative DGGE gels showing the prominent bands with the corresponding clade names. (PDF) [file pone.0103179.s002.pdf]

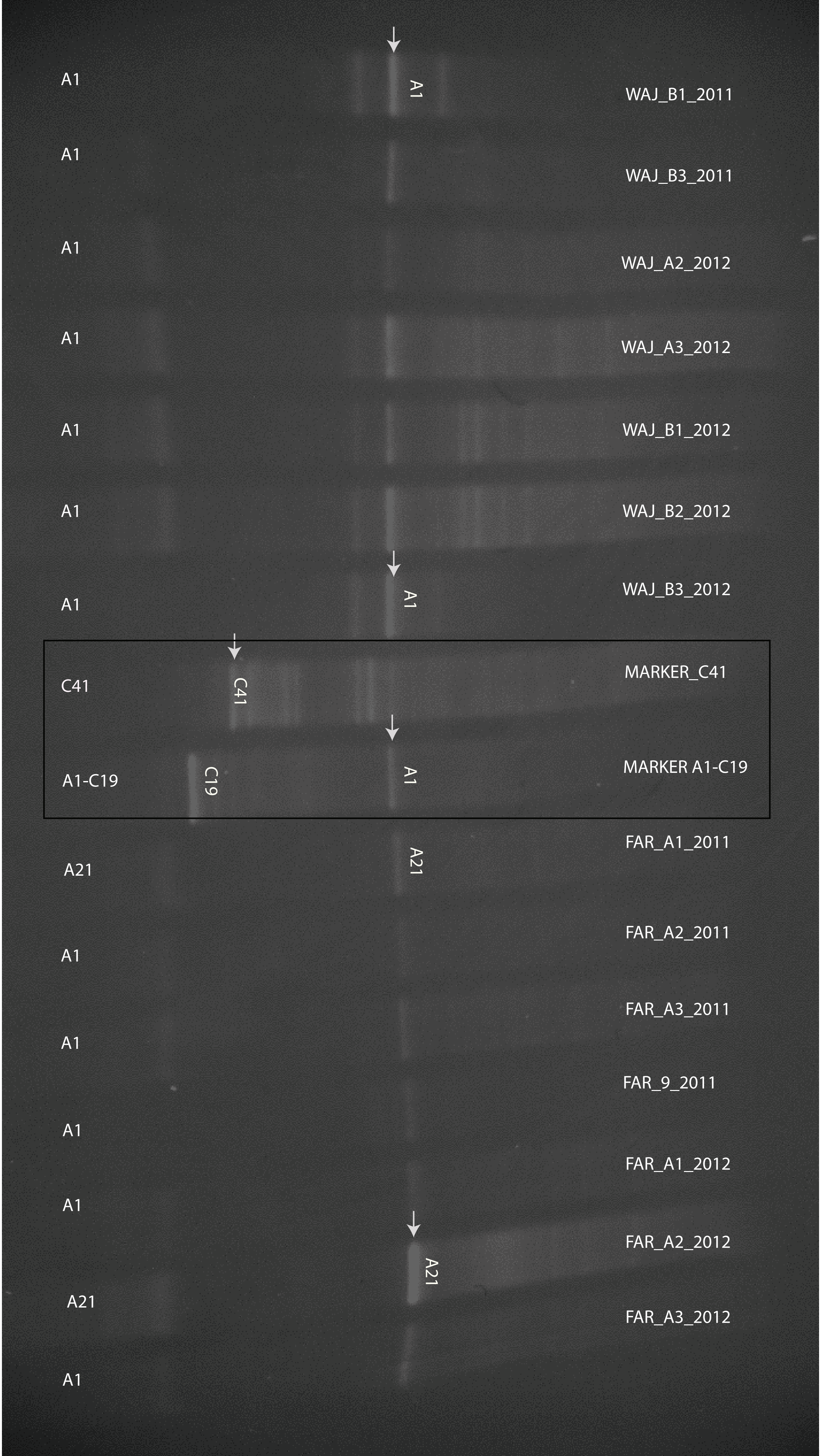
A1
A1
WAJ_B1_2011
A1
WAJ_B3_2011
A1
WAJ_A2_2012
A1
WAJ_A3_2012
A1
WAJ_B1_2012
A1
WAJ_B2_2012
A1
A1
WAJ_B3_2012
C41
C41
MARKER_C41
A1-C19
C19
A1
MARKER A1-C19
A21
A21
FAR_A1_2011
A1
FAR_A2_2011
A1
FAR_A3_2011
A1
FAR_9_2011
A1
FAR_A1_2012
A21
FAR_A2_2012
A21
FAR_A3_2012
A1

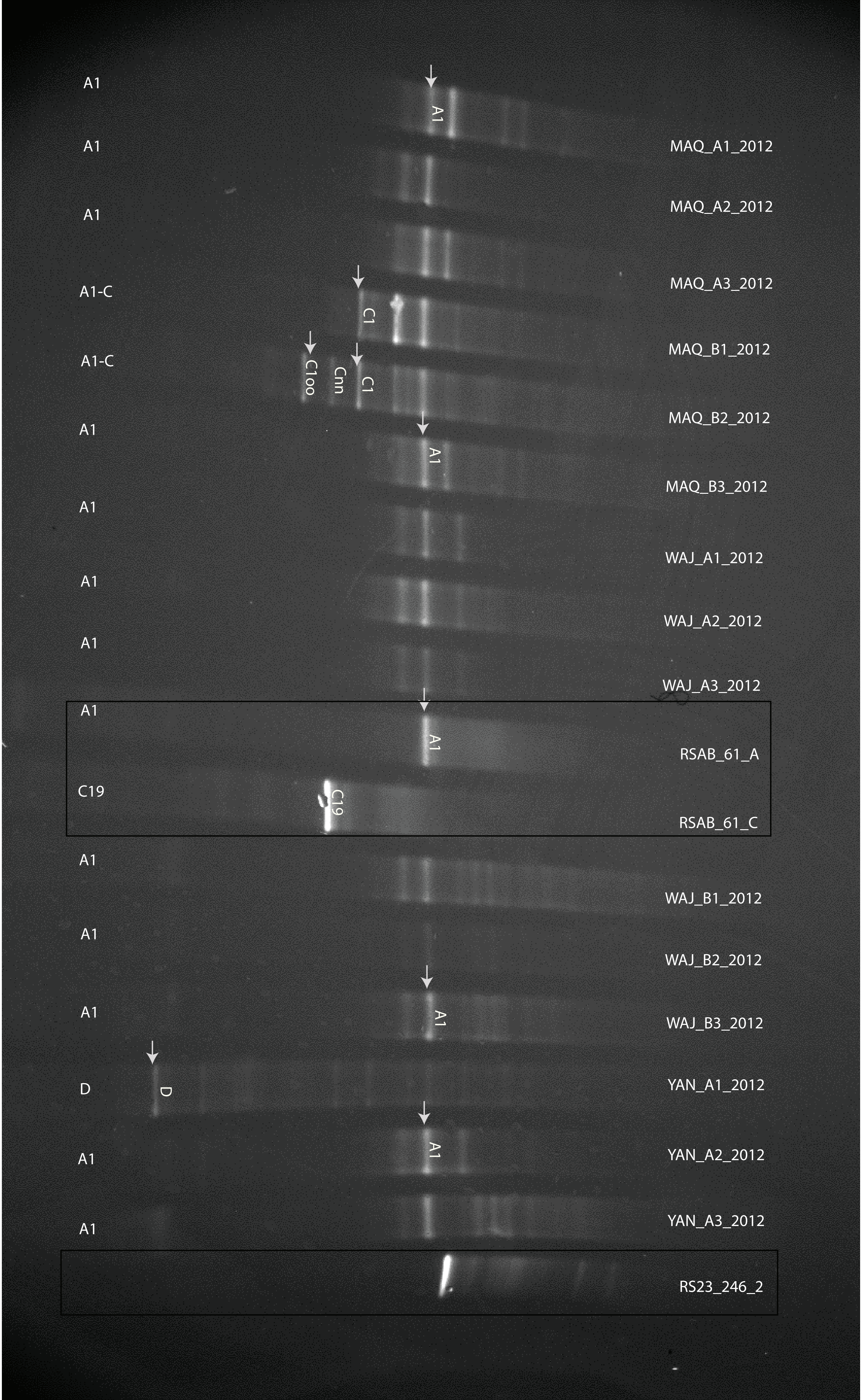
A1
A1
MAQ_A1_2012
A1
MAQ_A2_2012
A1
A1-C
C1
MAQ_A3_2012
A1-C
C1oo
Cnn
C1
MAQ_B1_2012
A1
A1
MAQ_B2_2012
A1
MAQ_B3_2012
A1
WAJ_A1_2012
A1
WAJ_A2_2012
A1
WAJ_A3_2012
A1
A1
RSAB_61_A
C19
C19
RSAB_61_C
A1
WAJ_B1_2012
A1
WAJ_B2_2012
A1
A1
WAJ_B3_2012
D
D
YAN_A1_2012
A1
A1
YAN_A2_2012
A1
YAN_A3_2012
RS23_246_2

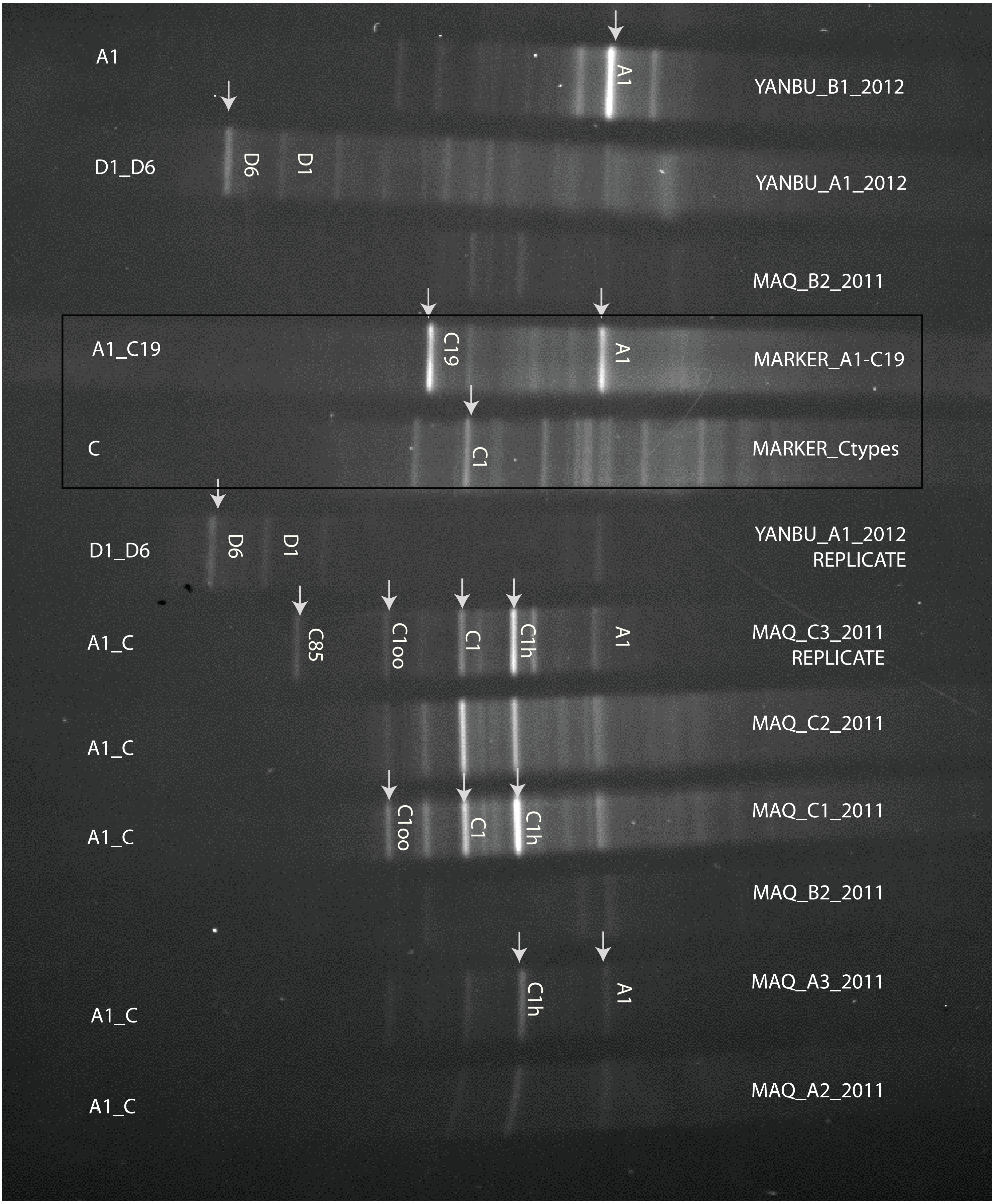
A1
A1
YANBU_B1_2012
D1_D6
D6
D1
YANBU_A1_2012
MAQ_B2_2011
A1_C19
C19
A1
MARKER_A1-C19
C
C1
MARKER_Ctypes
D1_D6
D6
D1
YANBU_A1_2012
REPLICATE
A1_C
C85
C1oo
C1
C1h
A1
MAQ_C3_2011
REPLICATE
A1_C
MAQ_C2_2011
A1_C
C1oo
C1
C1h
MAQ_C1_2011
MAQ_B2_2011
A1_C
C1h
A1
MAQ_A3_2011
A1_C
MAQ_A2_2011

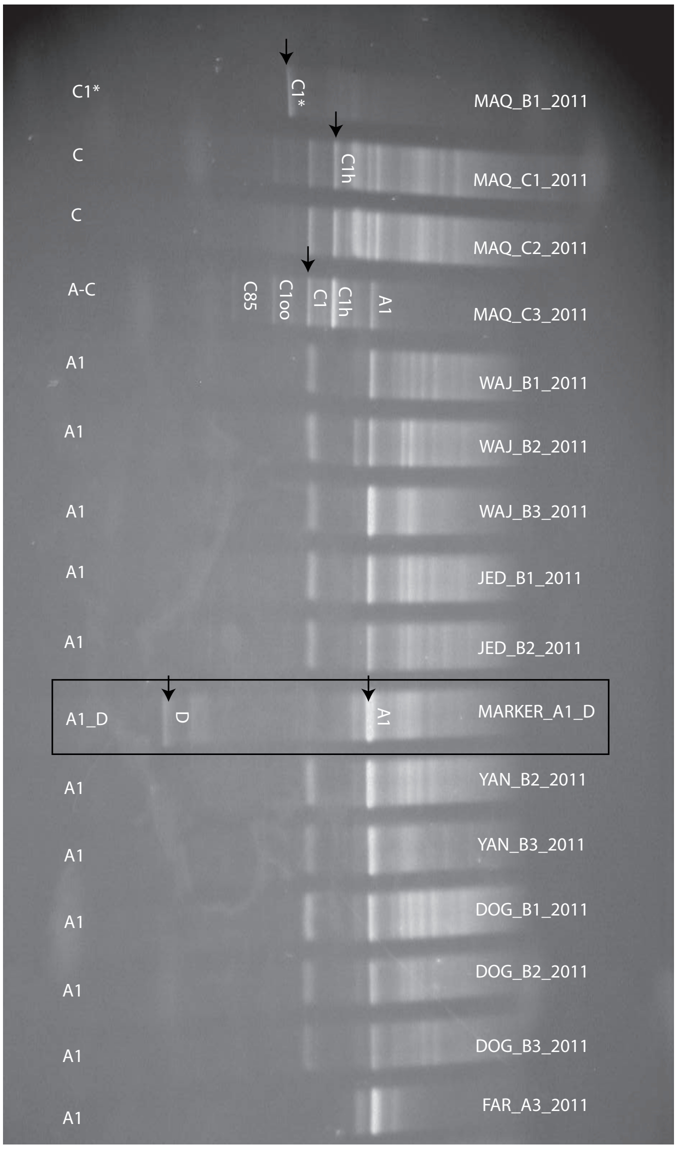
C1*
C1*
MAQ_B1_2011
C
C1h
MAQ_C1_2011
C
MAQ_C2_2011
A-C
C85
C1oo
C1
C1h
A1
MAQ_C3_2011
A1
WAJ_B1_2011
A1
WAJ_B2_2011
A1
WAJ_B3_2011
A1
JED_B1_2011
A1
JED_B2_2011
A1_D
D
A1
MARKER_A1_D
A1
YAN_B2_2011
A1
YAN_B3_2011
A1
DOG_B1_2011
A1
DOG_B2_2011
A1
DOG_B3_2011
A1
FAR_A3_2011
