# Supplementary material for: Spatio-Temporal Analyses of Symbiodinium Physiology of the Coral Pocillopora verrucosa along Large-Scale Nutrient and Temperature Gradients in the Red Sea
Source: PLoS One. 2014 Aug 19;9(8):e103179. doi: 10.1371/journal.pone.0103179 (PMC4138093; doi:10.1371/journal.pone.0103179)
Supplement: Figure S3 — P-I curves at all sites from N (top) to S (bottom) in September 2011 and March 2012. Mean± SE. (DOCX) [file pone.0103179.s003.docx]

Figure S2. **P-I curves** at all sites from N (top) to S (bottom) in September 2011 and March 2012. Mean± SE.
