# Supplementary material for: Spatio-Temporal Analyses of Symbiodinium Physiology of the Coral Pocillopora verrucosa along Large-Scale Nutrient and Temperature Gradients in the Red Sea
Source: PLoS One. 2014 Aug 19;9(8):e103179. doi: 10.1371/journal.pone.0103179 (PMC4138093; doi:10.1371/journal.pone.0103179)
Supplement: Table S2 — Zooxanthellae properties at all sites (1–6, North – South) in September 2011(Sep11) and March 2012 (Mar12). Photo-collecting pigments: sum of chlorophyll a and c2 and peridinin. N = 6. Mean (±SE). (DOCX) [file pone.0103179.s006.docx]

Table S2. **Zooxanthellae properties** at all sites (1 - 6, North – South) in September 2011(Sep11) and March 2012 (Mar12).

Photo-collecting pigments: sum of chlorophyll *a* and *c2* and peridinin. N=6. Mean (±SE).
