# Supplementary material for: Spatio-Temporal Analyses of Symbiodinium Physiology of the Coral Pocillopora verrucosa along Large-Scale Nutrient and Temperature Gradients in the Red Sea
Source: PLoS One. 2014 Aug 19;9(8):e103179. doi: 10.1371/journal.pone.0103179 (PMC4138093; doi:10.1371/journal.pone.0103179)
Supplement: Table S3 — Zooxanthellae performance at all sites (1–6, North – South) in September 2011(Sep11) and March 2012 (Mar12). Net photosynthesis and non-photochemical quenching (NPQ) at PAR 600 µmol photons m−2 s−1 (600), photo-collecting pigments (photo-coll. pig.). Maximum photosynthetic yield (Fv/Fm). N = 6. Mean (±SE). (DOCX) [file pone.0103179.s007.docx]

Table S3. Zooxanthellae performance at all sites (1 - 6, North – South) in September 2011(Sep11) and March 2012 (Mar12).

Net photosynthesis and non-photochemical quenching (NPQ) at PAR 600 µmol photons m^-2^ s^-1^ (600), photo-collecting pigments (photo-coll. pig.). Maximum photosynthetic yield (F_v_/F_m_). N=6. Mean (±SE).
