# Supplementary material for: Spatio-Temporal Analyses of Symbiodinium Physiology of the Coral Pocillopora verrucosa along Large-Scale Nutrient and Temperature Gradients in the Red Sea
Source: PLoS One. 2014 Aug 19;9(8):e103179. doi: 10.1371/journal.pone.0103179 (PMC4138093; doi:10.1371/journal.pone.0103179)
Supplement: Table S4 — Results of correlation and regression analyses. Dependent variables are the zooxanthellae characteristics density, photo-collecting pigments, and protein cell−1 and the ratio xanthophyll/chl a. The predictor variables (pred. var.) include the environmental parameters temperature (T), nutrients (N [flow*chl a]) and light (L [PAR]). Significant results are in bold (p>0.05). Analyses were conducted with complete data sets (season: all, site: all) and seasonal sub-sets (season: Sep or Mar, site: all) to differentiate between overall patterns across sites and seasons and pure geographic patterns (N-S), respectively. Furthermore, comparison of results between complete data sets and seasonal sub-sets allowed discriminating between seasonal and geographic responses to environmental variability. Since the correlation between temperature and nutrients or light was always significant within the seasonal sub-sets, no stepwise forward regression analyses were performed with these data sets, but partial correlation only. (DOCX) [file pone.0103179.s008.docx]

Table S4: Results of correlation and regression analyses.

Dependent variables are the zooxanthellae characteristics density, photo-collecting pigments, and protein cell^-1^ and the ratio xanthophyll / chl *a*. The predictor variables (pred. var.) include the environmental parameters temperature (**T**), nutrients (**N** [flow*chl *a*]) and light (**L** [PAR]). Significant results are in bold (p>0.05).

Analyses were conducted with complete data sets (season: all, site: all) and seasonal sub-sets (season: Sep or Mar, site: all) to differentiate between overall patterns across sites and seasons and pure geographic patterns (N-S), respectively. Furthermore, comparison of results between complete data sets and seasonal sub-sets allowed discriminating between seasonal and geographic responses to environmental variability. Since the correlation between temperature and nutrients or light was always significant within the seasonal sub-sets (Tab. 5 – cor. btw. pred. var.), no stepwise forward regression analyses were performed with these data sets, but partial correlation only.
